# Endocrine crosstalk in cardiovascular-kidney-metabolic syndrome-related heart failure: the fibroblast growth factor 23-Klotho axis and cardiometabolic therapy

**DOI:** 10.3389/fendo.2026.1933732

**Published:** 2026-09-08

**Authors:** Dan Sun, Xingjia Wang, He Wang, Zhiyi Shen, Xiangxu Meng, Yabin Zhou

**Affiliations:** 1 Graduate School, Heilongjiang University of Chinese Medicine, Harbin, Heilongjiang, China; 2 The First Affiliated Hospital of Heilongjiang University of Chinese Medicine, Harbin, Heilongjiang, China; 3 The First School of Clinical Medicine, Heilongjiang University of Chinese Medicine, Harbin, Heilongjiang, China

**Keywords:** cardiometabolic therapy, cardiovascular-kidney-metabolic syndrome, chronic kidney disease, diabetic kidney disease, FGF23, heart failure with preserved ejection fraction, Klotho, mineral metabolism

## Abstract

Heart failure arising in cardiovascular-kidney-metabolic (CKM) syndrome, hereafter referred to as CKM-related heart failure (CKM-HF), is increasingly understood as the cardiac expression of multisystem endocrine and metabolic stress rather than as an isolated myocardial disorder. In this narrative review, we examine how kidney, bone, adipose tissue, gut, pancreas, vasculature, and myocardium communicate through maladaptive hormonal and metabolic signals, with particular emphasis on heart failure with preserved ejection fraction (HFpEF). We position the fibroblast growth factor 23 (FGF23)-Klotho axis as a candidate organizing framework for mineral endocrine stress in diabetic kidney disease and chronic kidney disease. Human observational studies associate higher circulating FGF23 and altered Klotho biology with adverse cardiovascular phenotypes, whereas preclinical studies provide mechanistic plausibility for links to cardiac hypertrophy and fibrosis. However, current evidence does not establish that elevated FGF23 directly causes cardiac remodeling in humans. We examine insulin resistance, adipose dysfunction, RAAS activation, anemia, and gut-derived metabolites selectively as modifiers, competing pathways, or confounders that shape the interpretation of FGF23-Klotho biology in CKM-related heart failure. Established cardiometabolic therapies are discussed according to their proven clinical effects and as modifiers of the context in which FGF23 and soluble Klotho are interpreted; their efficacy is not considered evidence of FGF23-Klotho pathway engagement or mediation. An FGF23-Klotho-centered CKM-HF framework may support research phenotyping, prospective biomarker validation, and mechanism-based trial design. However, the proposed phenotype remains unvalidated, circulating FGF23 and soluble Klotho are not established for routine clinical use, and their incremental value beyond conventional kidney, mineral, cardiac, and imaging markers remains unknown.

## Introduction

Heart failure (HF) has traditionally been organized around cardiac structure, ejection fraction, and hemodynamic congestion. That framework remains clinically indispensable, yet it is incomplete for the growing population of patients whose HF develops in the setting of obesity, diabetes, chronic kidney disease (CKD), albuminuria, hypertension, systemic inflammation, and mineral metabolism disturbance. The American Heart Association Presidential Advisory on cardiovascular-kidney-metabolic (CKM) health formalized this multisystem view by defining CKM syndrome as the pathophysiological interaction among metabolic risk, kidney disease, and cardiovascular disease (1). The implication for HF is substantial: the failing heart is often the visible endpoint of endocrine and metabolic stress transmitted across organs rather than the sole origin of disease (2–5). HF with preserved ejection fraction (HFpEF) illustrates the need for this reframing. HFpEF is heterogeneous and frequently coexists with obesity, diabetes, CKD, hypertension, atrial fibrillation, anemia, and systemic inflammation. Contemporary reviews emphasize that HFpEF cannot be reduced to a single ventricular relaxation abnormality because many patients have microvascular inflammation, cardiometabolic congestion, skeletal muscle dysfunction, impaired chronotropic reserve, and renal sodium-handling abnormalities (6–9). In CKM syndrome, these abnormalities are not parallel coincidences. In this review, endocrine crosstalk refers to the bidirectional inter-organ exchange of hormonal and metabolic signals, through which dysfunction in one organ alters the biological function of another and is, in turn, modified by reciprocal feedback. This crosstalk links adipose tissue, kidney, bone, gut, pancreas, vasculature, and myocardium through pathways including adipokines, RAAS/MR signaling, FGF23-Klotho biology, inflammatory mediators, and gut-derived metabolites (10–14).

**Figure 1:**
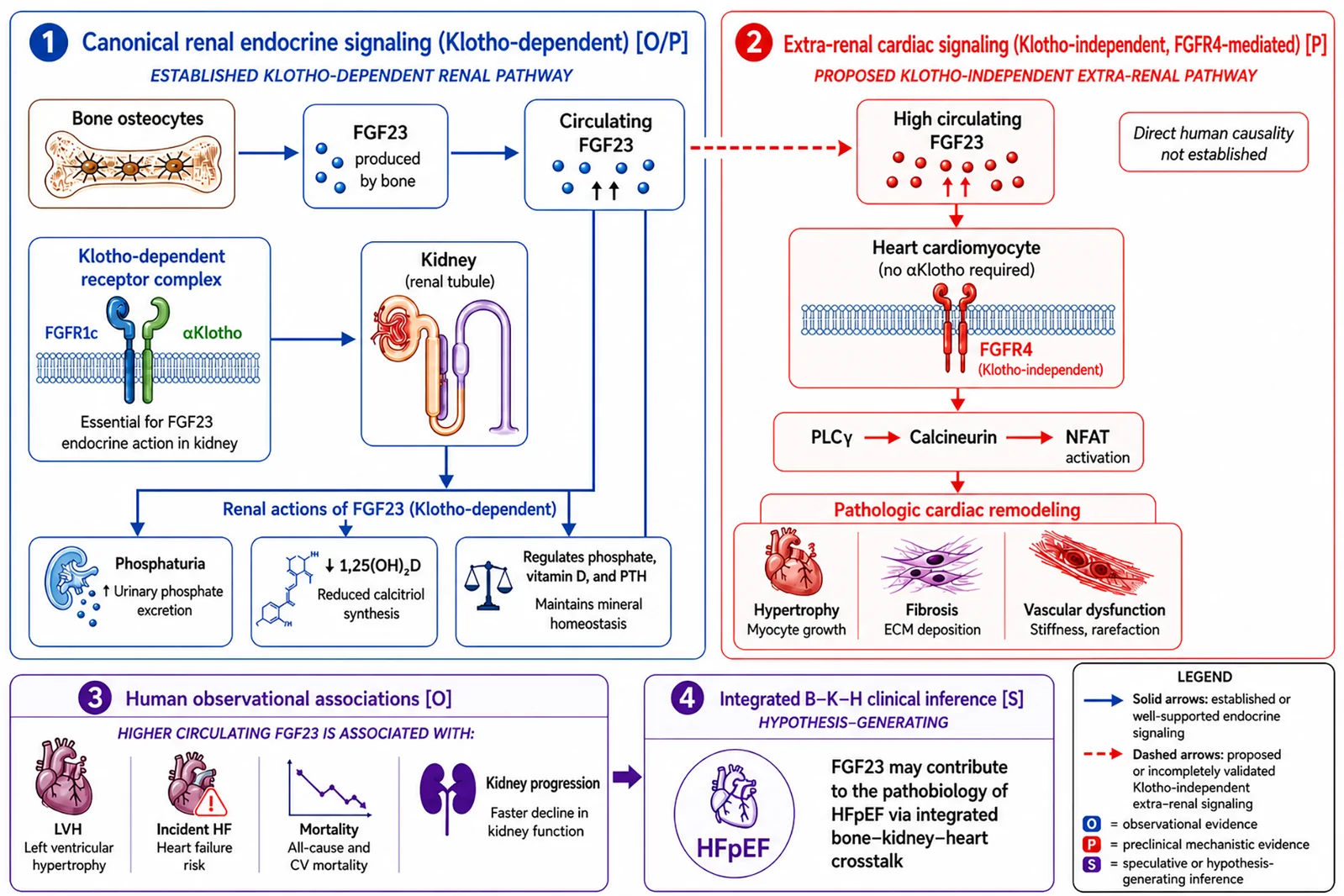
The FGF23-Klotho axis as a candidate bone-kidney-heart endocrine framework. The figure distinguishes two biologically related but evidentially distinct signaling pathways. The left pathway depicts the classical renal endocrine actions of bone-derived FGF23. In this canonical pathway, FGF23 acts predominantly on the kidney through FGFR1c in the presence of αKlotho, thereby promoting phosphaturia, suppressing 1,25(OH)_2_D, and contributing to regulation of phosphate, vitamin D, and parathyroid hormone balance. This renal pathway is relatively well established physiologically and is represented by solid arrows. The right pathway depicts proposed extra-renal cardiac signaling. In this pathway, high circulating FGF23 is hypothesized to act on the heart in a Klotho-independent manner through FGFR4-related signaling, with downstream activation of PLCγ/calcineurin-NFAT pathways and promotion of hypertrophic or fibrotic remodeling. This pathway is supported mainly by preclinical mechanistic evidence and is represented by dashed arrows. Direct causality in humans has not been established. The terminal grouping of hypertrophy, fibrosis, and vascular dysfunction is used for visual organization and should not be interpreted as indicating a single shared causal pathway. In particular, vascular dysfunction reflects broader mineral-endocrine and observational associations and is not presented as an established downstream consequence of cardiomyocyte FGFR4 signaling. Evidence labels indicate the dominant source and clinical directness of support for each component: O, human observational or translational evidence; P, preclinical mechanistic evidence; and S, speculative or hypothesis-generating inference. Thus, the canonical kidney pathway is supported mainly by O/P evidence, the FGFR4-mediated cardiac mechanism mainly by P evidence, and the extension from circulating FGF23-Klotho abnormalities to a distinct human heart-failure phenotype remains a hypothesis-generating clinical inference. Human studies currently support associations between higher circulating FGF23 and left ventricular hypertrophy, incident heart failure, mortality, and kidney progression, but these associations do not establish direct mediation of cardiac remodeling in patients.

This review treats the fibroblast growth factor 23 (FGF23)-Klotho axis as a candidate mechanistic bridge for understanding CKM-related HF, rather than as a single sufficient cause of the syndrome. FGF23 is a bone-derived hormone that regulates phosphate and vitamin D metabolism through kidney signaling that normally requires Klotho. In CKD and diabetic kidney disease (DKD), FGF23 rises early, soluble and membrane Klotho decline, phosphate and parathyroid hormone regulation become disturbed, and inflammatory and fibrotic pathways may become amplified (15, 16). Preclinical mechanistic studies have suggested that FGF23 may promote left ventricular hypertrophy (LVH) through direct cardiac signaling pathways, whereas human observational studies have associated higher circulating FGF23 concentrations with LVH and heart-failure risk in CKD populations (17, 18). These lines of evidence support the biological plausibility of an endocrine link between kidney dysfunction, mineral metabolism, and myocardial remodeling; however, observational associations do not establish causality or a clinically validated therapeutic threshold. As summarized in **Figure 1**, the review distinguishes the relatively established Klotho-dependent renal endocrine pathway from the predominantly preclinical, Klotho-independent FGFR4-mediated cardiac signaling pathway.

This review centers on a clinically oriented question: what role does FGF23-Klotho dysregulation play in CKM-related heart failure, particularly in CKD and DKD, and how should this axis be interpreted in relation to cardiac remodeling, heart-failure phenotypes, biomarker research, and the therapeutic context. Obesity, insulin resistance, RAAS activation, anemia, and gut-derived metabolites are considered only where they modify FGF23-Klotho biology, confound its associations with cardiovascular outcomes, define competing CKM-HF phenotypes, or influence the interpretation of treatment effects. The central aim is therefore not to provide a comprehensive review of all CKM endocrine pathways, but to position FGF23-Klotho biology within the specific clinical and mechanistic contexts required for appropriate evidence interpretation, phenotype-oriented research, and future pathway-directed studies, while recognizing that its current clinical actionability remains limited (1, 2, 15, 19–25).

## Search strategy and selection criteria

This structured narrative review was conducted to address the following clinically oriented question: how does inter-organ endocrine-metabolic crosstalk translate cardiovascular-kidney-metabolic syndrome into heart-failure risk, and what role does the FGF23-Klotho axis play within this network. The review was not prospectively registered. Because the original search log was not retained, all database searches were rerun during manuscript revision, and an aggregate revision-stage record was reconstructed to document database yields, duplicate removal, screening-flow counts, and eligibility criteria. PubMed/MEDLINE and the Web of Science Core Collection were searched from database inception to 18 July 2026. Targeted supplementary searches addressing phosphate/CKD-MBD interventions and sex-related heterogeneity (Q7–Q8 in **Supplementary Table 1**) were run on 25 July 2026, with publication-date coverage capped at 18 July 2026. Google Scholar was used as a supplementary source for forward citation tracking and for identifying potentially relevant publications not captured by the primary database searches. In addition, the reference lists of key guidelines, systematic reviews, clinical trials, and mechanistic studies were manually screened. The complete database-specific search strategies, including all Boolean operators, field tags, and the number of records retrieved from each search, are provided in **Supplementary Table 1**.

The search strategy was divided into complementary conceptual modules covering (1): cardiovascular-kidney-metabolic syndrome and heart-failure phenotypes (2); chronic kidney disease, diabetic kidney disease, and cardiorenal interactions (3); FGF23, Klotho, phosphate, parathyroid hormone, vitamin D, myocardial hypertrophy, and fibrosis (4); insulin resistance, adipose-tissue dysfunction, renin-angiotensin-aldosterone system activation, mineralocorticoid receptor signaling, and gut microbiota-derived metabolites; and (5) SGLT2 inhibitors, GLP-1 receptor agonists, incretin-based therapies, finerenone and other mineralocorticoid receptor antagonists, and weight-loss interventions.

Eligible publications included clinical guidelines, randomized controlled trials, prospective and retrospective cohort studies, case-control studies, analytical cross-sectional studies, systematic reviews and meta-analyses, human biomarker and imaging studies, and mechanistic experimental studies directly addressing CKM staging, heart-failure phenotyping, renal endocrine dysfunction, FGF23-Klotho biology, inter-organ signaling, or cardiometabolic therapy. Earlier publications were retained when they established fundamental biological mechanisms or clinical concepts.

Publications were excluded if they did not directly address CKM-related heart failure or a prespecified component of the endocrine-metabolic network; examined isolated phosphate metabolism without relevant renal or cardiovascular interpretation; reported microbiome associations without a kidney, metabolic, or heart-failure context; were available only as abstracts with insufficient methodological or outcome information; duplicated another publication or cohort report; or did not provide information relevant to the review question. The searches were performed by DS. Titles and abstracts were screened by XW and HW, with independent verification by YZ. Potentially relevant publications underwent full-text assessment. Disagreements were resolved through discussion with the other authors when necessary. The principal reasons for full-text exclusion were defined during the revision-stage screening process and are summarized in **Supplementary Figure 1**. Category-specific exclusion counts were not available because the original record-level screening log had not been retained. The searches identified 25,416 records from PubMed/MEDLINE, 25,690 records from the Web of Science Core Collection, and 1,200 additional records through Google Scholar, citation tracking, and reference-list screening. Because the original record-level screening log had not been retained, the number of duplicate records and the screening-flow counts were reconstructed during revision on the basis of the rerun database searches, overlap across databases and conceptual modules, and the final set of included publications. After removal of 18,642 duplicate records, 33,664 records underwent title and abstract screening, of which 319 publications were assessed in full text. Following exclusion of 212 full-text publications, 107 publications were included in the narrative review. The study-identification and screening process is shown in **Supplementary Figure 1**.

Because this study was designed as a structured narrative review rather than a systematic review or meta-analysis, the purpose of the search was to provide a transparent, reproducible, and clinically representative synthesis rather than an exhaustive quantitative summary of all eligible evidence. Evidence-generating publications were appraised using tools appropriate to their study designs, whereas narrative reviews, conceptual frameworks, and scientific statements were retained as contextual sources and were not permitted to independently determine the certainty of clinical conclusions.

## Risk-of-bias appraisal, certainty assessment, and evidence-tier assignment

Evidence-generating publications were independently appraised by two reviewers (DS and XW) using design-specific instruments. Randomized controlled trials were assessed at the outcome level using Cochrane RoB 2. Prognostic-factor studies were evaluated using QUIPS, whereas other cohort, case-control, analytical cross-sectional, and quasi-experimental studies were assessed using the corresponding JBI critical appraisal tools. Prediction-model studies were assessed using PROBAST. Systematic reviews and meta-analyses were evaluated using AMSTAR 2 or the JBI checklist for systematic reviews and research syntheses, as appropriate, with consideration of the risk-of-bias judgments for the underlying primary studies. Clinical practice guidelines and measurement recommendations were assessed using AGREE II or AGREE II-GRS. Animal experiments were assessed using the SYRCLE risk-of-bias tool. For *in-vitro* mechanistic studies, predefined reporting criteria addressing experimental controls, independent replication, blinding, sample-size or replicate justification, completeness of reporting, and validation across models were applied.

Narrative reviews, scientific frameworks, and conceptual publications were appraised only as contextual sources. SANRA was used where appropriate to assess the reporting quality of narrative reviews; however, these publications were not permitted to independently determine the certainty of a clinical or therapeutic conclusion. DS and XW resolved disagreements through discussion and consensus. Because the instruments use different domains and judgment categories, the results were not converted into a common numerical score. Consensus study-level judgments and the principal reasons for each judgment are presented in **Supplementary Table 2**.

Evidence-tier assignment was based on clinical directness and actionability after consideration of study design, risk of bias, consistency, and applicability. Tier 1 comprised evidence capable of informing current practice, generally supported by guideline-endorsed recommendations or randomized outcome evidence with at least moderate certainty. A publication was not assigned to Tier 1 solely because it was randomized; small trials, surrogate-outcome studies, and secondary analyses were classified as supportive evidence when they were insufficient to independently establish clinical actionability. Tier 2 comprised human observational, prognostic, biomarker, physiological, imaging, prediction-model, and supportive clinical evidence that informed association, risk stratification, or phenotyping but did not independently establish causality or treatment efficacy. Tier 3 comprised animal, cellular, assay-based, and early mechanistic evidence that supported biological plausibility but remained indirect to patient outcomes. Contextual sources were not independently tier-defining.

In a second stage, the certainty of the evidence supporting the major clinical and translational propositions was evaluated using a GRADE-informed framework. Risk of bias, inconsistency, indirectness, imprecision, and possible publication bias were considered for each proposition-specific body of evidence. Certainty was categorized as high, moderate, low, or very low. This assessment was applied to bodies of evidence rather than individual publications and was used to determine the strength of wording permitted in the manuscript. The proposition-level assessments are reported in **Supplementary Table 3**.

Of the 107 publications appraised in **Supplementary Table 2**, 18 narrative reviews, scientific frameworks, or conceptual reports were retained as contextual sources. The remaining 89 publications were assigned to evidence tiers: 48 Tier 1, 26 Tier 2, and 15 Tier 3. Tier 1 was dominated by randomized outcome trials, evidence-based guidelines, and diagnostic or measurement standards; Tier 2 consisted predominantly of observational cohorts, prognostic-factor studies, biomarker and physiological investigations, prediction models, and supportive subgroup or pooled analyses; and Tier 3 consisted mainly of animal, cellular, and mixed mechanistic studies.

**Figure 2:**
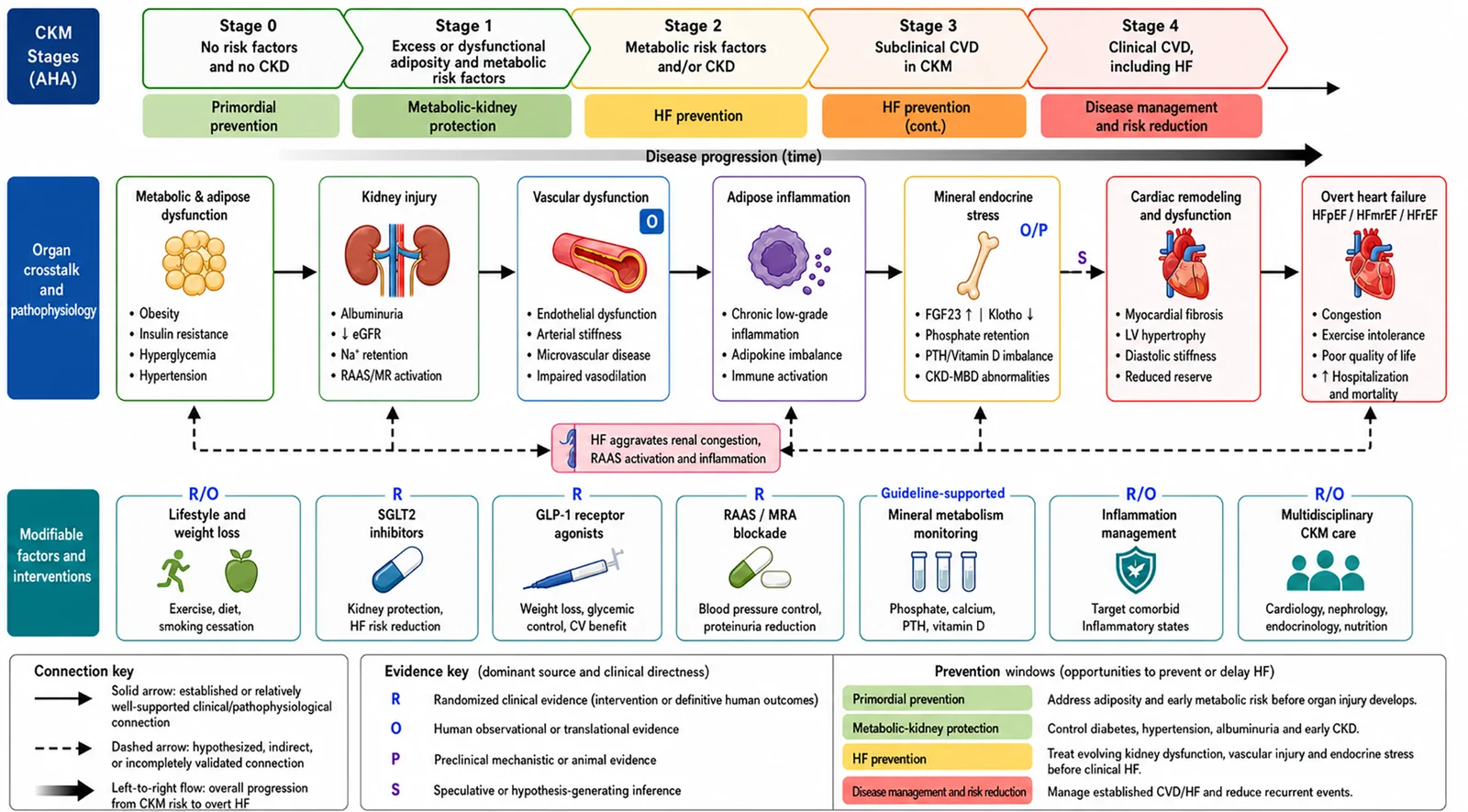
CKM syndrome reframes heart failure as a multisystem endocrine-metabolic disorder. The upper continuum illustrates CKM stages 0–4 and the corresponding opportunities for prevention and treatment. The middle pathway summarizes the predominant progression from metabolic risk, including obesity, type 2 diabetes, and hypertension, through kidney injury, vascular dysfunction, adipose inflammation, mineral-endocrine stress, and cardiac remodeling to clinically apparent HFpEF, HFmrEF, or HFrEF. This left-to-right arrangement is used for visual clarity and does not imply that all patients progress through an identical or strictly linear sequence. Inter-organ information flow is multidirectional. Metabolic and adipose dysfunction may promote inflammation, endothelial injury, hemodynamic stress, and kidney damage. Kidney injury contributes albuminuria, reduced eGFR, sodium and volume retention, RAAS/MR activation, and abnormalities in mineral-endocrine signaling. Vascular and adipose abnormalities further contribute to stiffness, endothelial dysfunction, inflammation, and impaired cardiac reserve. Mineral-endocrine stress, including higher circulating FGF23 and lower Klotho availability, may interact with these processes and contribute to cardiac remodeling, although direct FGF23-Klotho-specific progression to a distinct human heart-failure phenotype remains unproven. Established heart failure may in turn worsen renal congestion, RAAS activation, and systemic inflammation, creating a bidirectional cardiorenal feedback loop. Solid arrows indicate established or relatively well-supported clinical, epidemiological, or pathophysiological connections. Dashed arrows indicate hypothesized, indirect, or incompletely validated connections, particularly proposed FGF23-Klotho-specific links to cardiac remodeling. Evidence labels indicate the dominant evidence source and clinical directness: R, randomized clinical evidence; O, human observational or translational evidence; P, preclinical mechanistic evidence; and S, speculative or hypothesis-generating inference. Guideline- or consensus-supported clinical frameworks are identified separately and are not assigned an R label solely because they inform clinical practice. The prevention windows represent stages at which intervention may prevent or delay progression toward overt heart failure. Primordial prevention addresses adiposity and early metabolic risk before organ injury develops. Metabolic-kidney protection targets diabetes, hypertension, albuminuria, and early CKD to reduce downstream cardiorenal risk. HF prevention addresses evolving kidney dysfunction, vascular injury, endocrine-metabolic stress, and subclinical cardiovascular disease before clinical decompensation. Once overt cardiovascular disease or heart failure develops, management becomes increasingly phenotype-directed and emphasizes congestion control, guideline-supported HF therapy, and coordinated cardiovascular, kidney, and metabolic care.

**Figure 3:**
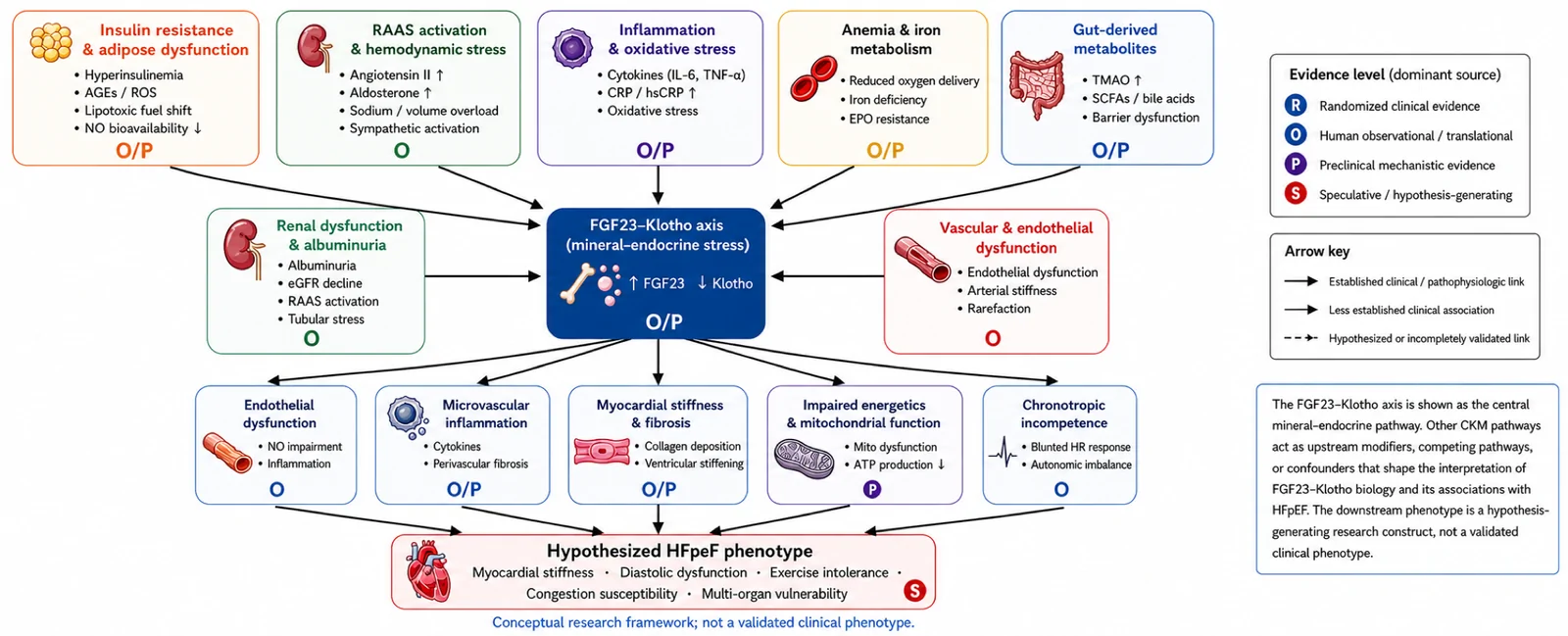
Parallel endocrine drivers of CKM-related HFpEF. The figure positions the FGF23-Klotho axis as the central mineral-endocrine pathway within a broader CKM network. Renal dysfunction, insulin resistance and adipose dysfunction, RAAS activation and hemodynamic stress, anemia and iron dysregulation, inflammation and oxidative stress, vascular dysfunction, and gut-derived metabolites are presented as upstream modifiers, competing pathways, or confounders rather than as evidence that FGF23-Klotho signaling independently mediates CKM-related heart failure. Arrows directed toward the central FGF23-Klotho box denote conceptual influence on pathway interpretation or association and do not necessarily imply direct molecular regulation of FGF23 or Klotho. These interacting pathways may converge on endothelial dysfunction, microvascular inflammation, myocardial stiffness and fibrosis, impaired energetics and mitochondrial function, and chronotropic incompetence. The downstream HFpEF pattern is therefore presented as a hypothesis-generating integration of multisystem abnormalities rather than as a prospectively validated causal sequence, clinical phenotype, or treatment-selection model. Solid arrows indicate established or relatively well-supported clinical or pathophysiological connections, whereas dashed arrows indicate proposed, indirect, or incompletely validated links. Evidence labels indicate the dominant source and clinical directness of support: R, randomized clinical evidence; O, human observational or translational evidence; P, preclinical mechanistic evidence; and S, speculative or hypothesis-generating inference. Combined labels indicate that different components of the same relationship are supported by different evidence types.

**Figure 4:**
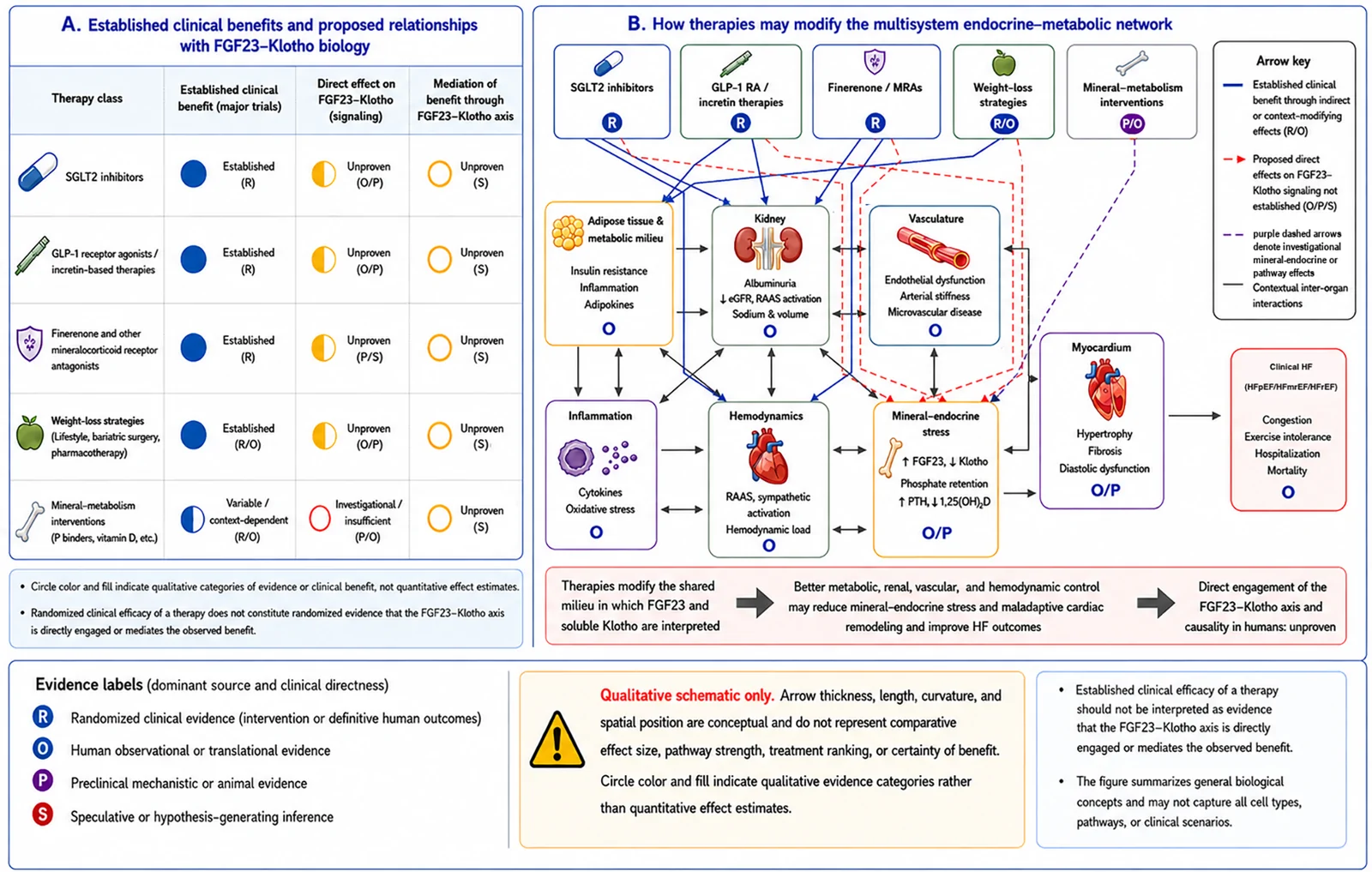
Cardiometabolic therapies as modifiers of inter-organ crosstalk. **(A)** provides a qualitative comparison of established clinical benefits and proposed relationships with FGF23-Klotho biology. SGLT2 inhibitors, GLP-1 receptor agonist/incretin-based therapies, finerenone and other mineralocorticoid receptor antagonists, and weight-loss strategies have established or supportive clinical benefits in appropriately selected populations, whereas their direct effects on FGF23-Klotho signaling and the mediation of clinical benefit through this axis remain unproven. Mineral-metabolism interventions are clinically indicated in selected CKD-MBD contexts, but pathway restoration and cardiovascular or heart-failure benefits remain investigational or insufficiently established. **(B)** illustrates how these therapies may modify the shared renal, adipose-metabolic, vascular, inflammatory, hemodynamic, and mineral-endocrine milieu in which FGF23 and soluble Klotho are interpreted. Solid blue arrows denote established clinical benefit through indirect or context-modifying effects; red dashed arrows denote proposed effects on FGF23-Klotho or mineral-endocrine signaling that have not been established; purple dashed arrows denote investigational mineral-endocrine or pathway effects; and gray arrows represent broader contextual inter-organ interactions. The connections shown are conceptual and are not intended to imply that every clinical benefit is mediated through the FGF23-Klotho axis. In particular, the gray connection between mineral-endocrine stress and the myocardium represents a broader pathophysiological relationship and should not be interpreted as evidence of established direct FGF23-Klotho-mediated cardiac causality in humans. Evidence labels indicate the dominant source and clinical directness of support: R, randomized clinical evidence; O, human observational or translational evidence; P, preclinical mechanistic evidence; and S, speculative or hypothesis-generating inference. Combined labels indicate that different components of the same relationship may be supported by different evidence types. For mineral-metabolism interventions, O/P or P/O labels describe the evidence informing mineral-endocrine pathway relationships; clinical mediation of cardiovascular or heart-failure benefit remains unestablished. Clinical HF in this schematic encompasses preserved, mildly reduced, and reduced ejection-fraction phenotypes. This figure is a qualitative conceptual schematic. Arrow thickness, length, curvature, and spatial position are used only for visual organization and do not represent comparative effect size, pathway strength, treatment ranking, or certainty of clinical benefit. Circle color and fill indicate qualitative categories rather than quantitative treatment effects. Randomized clinical efficacy of a therapy should not be interpreted as randomized evidence that the FGF23-Klotho axis is directly engaged or mediates the observed benefit.

To ensure consistency across the narrative synthesis and visual summaries, key claims in the main text, all main-text tables, and **Figures 1**–**4** were annotated using four standardized evidence labels: R, randomized clinical evidence; O, human observational or translational evidence; P, preclinical mechanistic evidence; and S, speculative or hypothesis-generating inference. In the narrative text, labels are provided at the first presentation of major mechanistic, biomarker, phenotypic, or therapeutic claims rather than after every sentence, to preserve readability. Combined labels are used when different components of a claim are supported by different evidence types. These labels indicate the source and clinical directness of the evidence rather than a formal numerical certainty score; proposition-level certainty is reported separately in **Supplementary Table 3**.

## CKM syndrome as a framework for heart failure

The CKM construct recognizes that metabolic risk factors, kidney dysfunction, and cardiovascular disease form a reinforcing continuum. The AHA advisory describes staged CKM health, ranging from metabolic risk without overt disease to established cardiovascular disease in the setting of kidney and metabolic abnormalities (1). This framework shifts attention from late-stage symptom management to upstream identification of HF risk. The AHA PREVENT equations further operationalize CKM health by incorporating cardiovascular, kidney, and metabolic variables into absolute risk assessment, while CKD guidance emphasizes combined eGFR-albuminuria staging for risk-based monitoring (26–31). As illustrated in **Figure 2**, the CKM framework should be interpreted as a staged, multisystem model in which established organ interactions coexist with candidate endocrine and translational links, thereby defining multiple prevention windows before overt heart failure develops. The timeline emphasizes prevention windows because CKM stage progression is not merely a descriptive classification; it is a practical prompt to treat albuminuria, obesity, diabetes, hypertension, and mineral stress before irreversible myocardial remodeling develops (1, 26, 27).

A useful feature of CKM staging is that it separates disease burden from clinical presentation. Two patients may have similar left ventricular ejection fraction but very different upstream biology: one dominated by prior infarction and neurohormonal remodeling, another by obesity, albuminuria, insulin resistance, and mineral endocrine stress. Conversely, patients with similar diabetes or CKD stage may diverge in HF risk depending on vascular stiffness, adipose inflammation, sex, age, access to therapy, and the pace of kidney decline. CKM staging therefore encourages clinicians and investigators to follow trajectories rather than isolated diagnoses (1, 6, 28). For HF, the CKM framework is particularly valuable because classic risk factors rarely act in isolation. Albuminuria, reduced estimated glomerular filtration rate (eGFR), diabetes, adiposity, hypertension, and inflammation collectively predict cardiovascular events and HF hospitalization. Albuminuria is now recognized not only as a kidney marker but also as a systemic vascular and cardiometabolic risk signal (32). The 2024 KDIGO CKD guideline reinforces the importance of combined eGFR and albuminuria staging, risk-based monitoring, and integrated management of CKD complications (4, 5, 28–31).

CKM syndrome also helps distinguish HFpEF from HFrEF without treating them as entirely separate diseases. HFrEF is often dominated by myocardial injury, neurohormonal activation, and adverse ventricular remodeling, whereas HFpEF more frequently reflects systemic metabolic inflammation, obesity, renal congestion, endothelial dysfunction, and myocardial stiffness (3, 7–14). However, CKM biology crosses ejection-fraction categories. Diabetes, CKD, albuminuria, and related systemic abnormalities contribute to HF risk through multiple established pathways, whereas higher circulating FGF23 has been associated with both preserved- and reduced-ejection-fraction heart-failure phenotypes in observational studies. For FGF23-Klotho biology, EF-specific evidence remains mainly observational or translational, with MESA, PREVEND, and CRIC suggesting subtype-relevant associations rather than definitive treatment thresholds (20, 23, 25, 33–37).

A CKM approach therefore supports an organ-network model of HF. The clinical phenotype emerges from bidirectional feedback: kidney dysfunction activates endocrine and mineral stress; adipose tissue promotes inflammation and altered natriuretic peptide biology; hyperglycemia and insulin resistance impair vascular and myocardial metabolism; gut metabolites influence inflammation and thrombosis; and the myocardium responds with hypertrophy, fibrosis, stiffness, and impaired reserve. This network is the substrate on which modern cardiometabolic therapies act (1, 6, 28, 32). Kidney-protective trials and CKD guidance define the clinical context for this network (38–40). Mechanistic and clinical studies of diabetic cardiomyopathy describe complementary myocardial and metabolic pathways (41–43). Large cardiovascular-outcome trials of GLP-1 receptor agonists and SGLT2 inhibition, together with lifestyle and semaglutide studies in obesity-related HFpEF, further define the relevant therapeutic context (44–51).

The framework also clarifies why CKM-related HF is not synonymous with cardiorenal syndrome. Cardiorenal syndrome often describes the bidirectional interaction between heart and kidney failure, especially in acute or chronic decompensation. CKM syndrome is broader: it includes metabolic and endocrine drivers that precede overt HF, and it recognizes obesity, diabetes, albuminuria, kidney dysfunction, and vascular injury as a shared disease field. The FGF23-Klotho axis fits this broader model because it is not only a renal complication but also a bone-derived endocrine response to kidney and mineral stress (1, 15, 16). This distinction has practical implications for both clinical care and trial design. Clinically, the CKM-HF framework extends attention beyond the management of established heart-kidney decompensation to the earlier identification and treatment of obesity, diabetes, albuminuria, declining eGFR, hypertension, and mineral-endocrine stress before overt heart failure develops. It does not replace standard cardiorenal assessment of congestion, kidney function, and treatment safety, but broadens the window for prevention and coordinated cardiovascular, kidney, and metabolic care. In clinical trials, CKM-HF should not be treated as a biologically homogeneous condition. Eligibility criteria, stratification, and biomarker substudies should prespecify CKM stage, kidney phenotype, including eGFR and albuminuria, adiposity or diabetes status, ejection-fraction category, and relevant treatment exposure, while outcomes should capture both heart-failure and kidney events. Such phenotype enrichment may reduce mechanistic heterogeneity and clarify which patient subgroups derive benefit (1, 6, 20, 23, 28). Within this broader CKM framework, FGF23-Klotho is introduced only as a mineral-endocrine component; its canonical renal biology, Klotho-independent FGFR4/calcineurin-NFAT signaling, human associations, and therapeutic evidence are examined in the following dedicated section.

## FGF23-Klotho biology in DKD/CKD-related heart failure: mechanistic and clinical evidence

FGF23-Klotho biology is best viewed as a candidate organizing framework within CKM-related heart failure because abnormalities in mineral metabolism, renal endocrine function, inflammation, and cardiac structure frequently coexist in CKD and DKD. Rising FGF23, declining Klotho expression, phosphate burden, and inflammatory activation may be detectable before overt heart failure becomes clinically manifest. Human studies support associations among these abnormalities, LVH, heart-failure risk, and adverse kidney outcomes, whereas experimental studies provide biological plausibility for several potential signaling pathways. FGF23 may therefore have dual research roles as an associated biomarker of CKD-related mineral and cardiovascular stress and as a potential mediator of LVH. However, the mediator interpretation is supported mainly by preclinical evidence, and whether elevated FGF23 is directly causal, represents an adaptive response that becomes maladaptive in selected contexts, or primarily reflects underlying kidney and mineral stress remains unresolved in humans (15–18, 28, 52–61).

### Preclinical mechanistic evidence [P]

Animal and cellular studies provide mechanistic support for a potential link between FGF23-Klotho dysregulation and cardiac remodeling. Experimental studies have shown that sustained FGF23 exposure may activate Klotho-independent FGFR4-related signaling and downstream calcineurin-NFAT pathways, thereby promoting hypertrophic gene programs in preclinical models (17, 56). These studies support a possible direct myocardial pathway that is distinct from canonical renal FGF23 signaling, which normally requires Klotho.

Preclinical evidence also suggests that the cardiovascular effects associated with FGF23-Klotho dysregulation may extend beyond hypertrophy. FGF23-related signaling may intersect with fibroblast activation, oxidative stress, inflammatory pathways, vitamin D suppression, and mineral-metabolism disturbance, whereas Klotho deficiency may contribute to phosphate dysregulation, vascular calcification, oxidative injury, and cardiovascular vulnerability (15–17, 19, 52–56, 62–66). Experimental FGF23 neutralization studies further illustrate the complexity of this axis, because suppression of FGF23 may improve selected biochemical abnormalities while aggravating phosphate retention or other adverse outcomes (55).

These findings provide biological plausibility for a bone-kidney-heart endocrine pathway but remain indirect to human clinical outcomes. They do not establish that FGF23 directly causes cardiac hypertrophy or fibrosis in patients, nor do they demonstrate that FGFR4 inhibition, FGF23 suppression, or Klotho restoration improves clinical heart-failure outcomes.

### Human observational and translational evidence [O]

Human evidence regarding the FGF23-Klotho axis remains predominantly observational and translational. Cross-sectional CKD data have associated higher circulating FGF23 concentrations with LVH, supporting a relationship between mineral endocrine stress and structural heart disease (18). Prospective cohort studies have subsequently extended these observations to clinical outcomes. In MESA, FGF23 was evaluated in relation to heart failure with reduced versus preserved ejection fraction; PREVEND and CRIC further examined associations with incident heart failure and heart-failure subtype (33–35). Other CKD cohorts have associated higher circulating FGF23 with cardiovascular events, mortality, and kidney-disease progression (57–60).

Collectively, these studies suggest that higher circulating FGF23 may identify patients with greater renal-mineral and cardiovascular risk. However, residual confounding by kidney function, phosphate balance, inflammation, anemia, frailty, and medication exposure, together with assay heterogeneity and possible reverse causation, limits causal interpretation. Higher circulating FGF23 should therefore be interpreted as an associated risk or phenotype marker rather than as evidence of a clinically validated causal pathway or treatment threshold.

These observations motivate investigation of a candidate mineral-endocrine-enriched HFpEF pattern characterized by CKD or DKD, higher FGF23, lower soluble Klotho, and cardiac structural or functional abnormalities. However, this combination is nonspecific and has not been prospectively validated as a distinct phenotype or endotype. Soluble Klotho may provide candidate information on renal endocrine reserve, but assay heterogeneity and the absence of validated thresholds limit interpretation. FGF23 and Klotho should therefore be evaluated together with eGFR, albuminuria, phosphate, PTH, vitamin D status, anemia, medication exposure, and cardiac imaging rather than in isolation (15, 16, 28, 32–35, 66).

### Randomized clinical evidence and therapeutic status [No direct R evidence; therapeutic implications remain S]

Randomized evidence has not established that direct modification of FGF23, FGFR4, or Klotho improves heart-failure outcomes, and no validated threshold identifies patients for pathway-directed treatment. Current randomized evidence instead supports established cardiometabolic and kidney-protective therapies for their studied indications, without demonstrating that FGF23-Klotho modification mediates their benefits. Direct pathway-directed strategies therefore remain investigational.

## CKM modifiers of FGF23-Klotho signaling and interpretation

FGF23-Klotho biology does not operate in isolation. Its circulating concentrations, biological interpretation, and association with heart-failure phenotypes are influenced by kidney function, glycemic and adipose-metabolic stress, RAAS activation, anemia, inflammation, medication exposure, and gut-derived metabolites. Detailed FGF23-Klotho physiology and cardiac signaling mechanisms are presented in the preceding dedicated section and are not repeated here. The following section therefore does not provide a comprehensive review of these broader CKM pathways. Instead, it considers them selectively as upstream regulators, competing mechanisms, confounders, or phenotype modifiers relevant to the interpretation of the FGF23-Klotho axis. **Table 1** summarizes these relationships, with the FGF23-Klotho axis positioned as the central mineral-endocrine pathway and the remaining domains presented as contextual modifiers.

**Table 1:** CKM modifiers of FGF23-Klotho signaling, interpretation, and heart-failure phenotyping: evidence type and implications.

Pathway or domain	Principal source organs or systems	Representative mediators or markers	Relationship to FGF23-Klotho signaling and interpretation	Most relevant CKM-HF phenotype	Evidence type and directness	Clinical or research implications
Bone-kidney-heart FGF23-Klotho axis	Bone, kidney, parathyroid gland, myocardium, and vasculature	FGF23, membrane and soluble Klotho, phosphate, PTH, 25(OH)D, 1,25(OH)_2_D, and FGFR4-related signaling	Central mineral-endocrine pathway. Canonical renal FGF23-Klotho signaling regulates phosphate and vitamin D homeostasis. Human studies associate higher FGF23 and altered Klotho biology with adverse cardiovascular phenotypes, whereas direct FGFR4-related cardiac signaling is supported mainly by preclinical studies.	CKD/DKD-related HF; candidate mineral-endocrine-enriched HFpEF; CKD-associated HF across ejection-fraction categories	O/P; S for the proposed clinical phenotype and therapeutic targeting. Human associations are O; direct cardiac mechanisms are P; no R evidence establishes direct pathway-targeted HF benefit.	Interpret biomarkers with kidney function and mineral parameters; support mechanistic research and trial enrichment; avoid assuming human causality, validated phenotype status, or routine FGF23/Klotho-directed treatment.
Renal dysfunction and phosphate burden	Glomeruli, renal tubules, renal interstitium, and systemic vasculature	eGFR, albuminuria, phosphate, PTH, vitamin D metabolites, Klotho, FGF23, and uremic toxins	Reduced nephron function, tubular injury, phosphate burden, and loss of renal Klotho are major upstream determinants. Kidney dysfunction also confounds associations between circulating FGF23 and cardiovascular outcomes.	Albuminuric DKD; CKD-HF; cardiorenal HF; HFpEF or HFrEF with declining eGFR	O/P. CKD-HF risk and eGFR/albuminuria associations are O; modification of FGF23-Klotho biology and phosphate handling is supported by O/P evidence.	Interpret FGF23 and Klotho according to eGFR, albuminuria, phosphate, PTH, and vitamin D status; prioritize longitudinal measurements.
Insulin resistance and hyperglycemia	Pancreas, liver, skeletal muscle, adipose tissue, kidney, and vasculature	Insulin resistance, hyperglycemia, advanced glycation end products, oxidative stress, inflammatory mediators, and albuminuria	Diabetes accelerates DKD, tubular injury, albuminuria, inflammation, and nephron loss, indirectly altering FGF23 and Klotho. Glycemic and renal disease severity may confound observed FGF23-HF associations.	T2D-related HF; DKD-associated HF; metabolically enriched HFpEF	O/P; S for a direct FGF23-Klotho interaction. Diabetes-HF and DKD risk are supported by O evidence; direct axis modification remains inferred.	Account for glycemic burden, DKD severity, kidney function, and treatment exposure in biomarker analyses.
Adipose dysfunction and obesity	Visceral and epicardial adipose tissue, vasculature, skeletal muscle, and kidney	Leptin, adiponectin, inflammatory cytokines, free fatty acids, plasma-volume expansion, and altered natriuretic-peptide biology	Obesity can produce a competing HFpEF pathway through inflammation, congestion, endothelial dysfunction, and impaired reserve. Its direct influence on FGF23-Klotho signaling is insufficiently defined.	Obesity-related HFpEF; CKM-HFpEF with inflammation and congestion	O/P for the obesity-HFpEF pathway; R for selected obesity-directed treatment outcomes; S for FGF23-Klotho mediation.	Distinguish obesity-dominant from kidney/mineral-endocrine-enriched HFpEF; do not infer axis mediation from weight-loss or incretin benefits.
RAAS/mineralocorticoid signaling and anemia	Kidney, adrenal gland, vasculature, myocardium, sympathetic nervous system, and hematopoietic system	Angiotensin II, aldosterone, sodium retention, oxidative stress, inflammation, hemoglobin and iron status	RAAS/MR activation and anemia amplify remodeling, congestion, and impaired oxygen delivery and may confound FGF23-outcome associations. Direct pathway interaction remains incompletely defined.	CKD-HF; cardiorenal HF; HFpEF/HFrEF with albuminuria, anemia, or congestion	O/P for pathway interaction; R for established RAAS/MR-directed outcomes; S for FGF23-Klotho mediation.	Adjust for RAAS-blocking therapy, hemoglobin, iron status, inflammation, and volume status; do not treat established RAAS/MR efficacy as evidence of FGF23-Klotho mediation.
Gut-derived metabolites	Gut microbiota, intestinal barrier, liver, kidney, immune system, and vasculature	TMAO, SCFAs, bile acids, endotoxin-related pathways, indoles, and p-cresol	Gut-derived metabolites may influence renal injury and systemic inflammation, but direct human interactions with FGF23 or Klotho remain insufficiently defined and strongly depend on diet and kidney clearance.	CKM-HF with CKD, obesity, diabetes, or diet-sensitive risk	O/P for cardiovascular and HF associations; S for direct interaction with FGF23-Klotho signaling and therapeutic relevance.	Retain as contextual modifiers and potential confounders; use for exploratory research rather than routine stratification.

Evidence labels: R, randomized clinical evidence; O, human observational or translational evidence; P, preclinical mechanistic evidence; S, speculative or hypothesis-generating inference. Combined labels indicate that different components of the same pathway are supported by different evidence types. Labels denote study type and clinical directness rather than effect size or formal certainty; detailed risk-of-bias and certainty assessments are provided in **Supplementary Tables 2, S3**.

CKM, cardiovascular-kidney-metabolic; HF, heart failure; HFpEF, heart failure with preserved ejection fraction; HFrEF, heart failure with reduced ejection fraction; CKD, chronic kidney disease; DKD, diabetic kidney disease; eGFR, estimated glomerular filtration rate; FGF23, fibroblast growth factor 23; PTH, parathyroid hormone; RAAS, renin-angiotensin-aldosterone system; MR, mineralocorticoid receptor; TMAO, trimethylamine N-oxide; SCFAs, short-chain fatty acids.

### Insulin resistance, hyperglycemia, and adipose dysfunction

Insulin resistance, hyperglycemia, and adipose dysfunction are relevant to this review primarily because they shape the renal and inflammatory environment in which FGF23-Klotho abnormalities develop and are interpreted. Diabetes accelerates albuminuria, tubular injury, inflammation, and loss of nephron reserve, all of which may contribute to rising FGF23 and declining Klotho before overt hyperphosphatemia develops. Glycemic burden, obesity, and systemic inflammation may also confound associations between FGF23 and adverse cardiac phenotypes and should therefore be considered in observational analyses and biomarker studies (6, 28, 32, 41–43).

Human observational and mechanistic evidence [O/P]. Adipose dysfunction also defines an important competing HFpEF phenotype. Visceral and epicardial adiposity, altered natriuretic-peptide biology, plasma-volume expansion, and systemic inflammation may produce HFpEF independently of mineral-endocrine stress. Consequently, obesity-dominant HFpEF should be distinguished from CKD/mineral-endocrine-enriched HFpEF when interpreting FGF23, Klotho, LVH, or fibrosis findings. Randomized clinical evidence [R]; FGF23-Klotho mediation [S]. Randomized benefits of semaglutide in obesity-related HFpEF establish adiposity as a modifiable clinical pathway in that selected phenotype, but they do not demonstrate that FGF23-Klotho modulation mediates the benefit (9–13, 23, 48–51).

### Renal dysfunction, RAAS/MR activation, and anemia as contextual modifiers

Contextual evidence [O/P]. Renal dysfunction is considered here primarily as an upstream determinant and major confounder of FGF23-Klotho measurements rather than as a second description of axis biology. Declining nephron function, albuminuria, tubular injury, inflammation, and phosphate burden can alter circulating FGF23 and Klotho while independently increasing heart-failure risk. These abnormalities also coexist with sodium retention, RAAS activation, anemia, and uremic toxicity, each of which independently contributes to heart-failure risk (28–32, 39, 40). In this context, RAAS activation and anemia should be interpreted as interacting or competing pathways rather than as separate core themes of the review. They may amplify cardiac remodeling and HF symptoms while also confounding associations between FGF23 and cardiovascular outcomes. Future FGF23-HF studies should therefore account for eGFR, albuminuria, phosphate, PTH, vitamin D, hemoglobin and iron status, RAAS-blocking therapy, inflammation, and volume status. This approach helps distinguish mineral-endocrine signaling from the broader consequences of CKD.

### Gut-derived metabolites as hypothesis-generating contextual modifiers

Preclinical mechanistic evidence [P]. Intestinal-barrier dysfunction, microbial dysbiosis, and gut-derived metabolites may influence renal injury, systemic inflammation, endothelial dysfunction, thrombosis, and metabolic signaling. Experimental evidence suggests that TMAO may promote vascular inflammation and endothelial injury, whereas short-chain fatty acids and bile acids may exert context-dependent effects through intestinal-barrier integrity, immune signaling, and endocrine receptors. These mechanisms provide biological plausibility for an indirect gut–kidney–cardiovascular pathway but do not establish direct regulation of FGF23 or Klotho.

Human observational and translational evidence [O]. Higher circulating TMAO has been associated with cardiovascular risk and adverse outcomes in heart-failure cohorts. However, circulating TMAO is strongly influenced by diet, microbial production, hepatic oxidation, kidney clearance, and medication exposure, which limits causal interpretation. Evidence concerning short-chain fatty acids, bile-acid profiles, and microbiome composition in CKM-related heart failure remains heterogeneous and insufficiently standardized, and no microbial or metabolite signature has been prospectively validated as an FGF23-Klotho-associated phenotype.

Direct-axis and therapeutic implications [S; no R evidence]. Whether gut-derived metabolites directly modify FGF23 production, Klotho expression, or FGF23-related cardiac signaling in human CKM-related heart failure remains unknown. Within the evidence reviewed, no microbiome-directed or metabolite-targeted intervention has demonstrated improvement in CKM-related heart-failure outcomes through modification of the FGF23-Klotho axis. Gut-derived metabolites are therefore considered hypothesis-generating contextual modifiers and potential confounders rather than a coequal mechanistic pathway or clinically actionable target in this review (67–77).

Framework-level inference [S]. **Figure 3** illustrates how these contextual modifiers may converge with kidney and mineral stress on endothelial dysfunction, fibrosis, myocardial stiffness, and a proposed mineral-endocrine-enriched HFpEF pattern. This figure represents a hypothesis-generating research framework rather than a validated causal sequence, diagnostic phenotype, or treatment-selection model. Preclinical evidence that FGF23/FGFR4-mediated left ventricular hypertrophy can be reversed further supports mechanistic investigation without establishing human therapeutic efficacy (61).

## Therapeutic implications for FGF23-Klotho biology in CKM-related heart failure

Established cardiometabolic therapies should be discussed according to the clinical outcomes directly evaluated in randomized trials. Their cardiovascular and kidney benefits do not, by themselves, provide indirect evidence that the FGF23-Klotho axis is clinically relevant, therapeutically modifiable, or involved in mediating treatment response. **Table 2** summarizes the established clinical efficacy, direct FGF23-Klotho evidence, and mediation status of the major cardiometabolic therapies discussed in this review. **Figure 4** presents these relationships visually, emphasizing that established therapies modify renal, metabolic, hemodynamic, and inflammatory context rather than acting as validated FGF23-Klotho-directed interventions. In this review, SGLT2 inhibitors, GLP-1 receptor agonists, and finerenone are therefore included primarily to define the renal, metabolic, hemodynamic, and inflammatory context in which FGF23 and soluble Klotho are measured and interpreted. Claims of direct pathway modification require therapy-specific evidence of changes in FGF23 or soluble Klotho, whereas claims of mediation require evidence that such changes account for subsequent clinical benefit. These levels of evidence should not be inferred from outcome efficacy alone.

**Table 2:** Clinical efficacy, direct FGF23-Klotho evidence, and mediation status of established and investigational therapies in CKM-related heart failure.

Therapy	Established clinical evidence	Direct evidence on FGF23/Klotho	Evidence of mediation	Interpretation for this review	Representative evidence
SGLT2 inhibitors	R: Established HF and kidney benefits in indicated HF and CKD populations.	Limited O/P: Short-term changes in phosphate-regulatory physiology and circulating FGF23 have been reported; direct soluble Klotho effects remain insufficiently characterized.	S: Not established. No clinical mediation analysis has shown that FGF23 or soluble Klotho changes account for improved HF or kidney outcomes.	Clinical benefit validates SGLT2 inhibition for established indications, not FGF23-Klotho pathway engagement or mediation.	Outcome trials: (20, 22, 25, 38); therapy-specific mechanistic studies where cited.
GLP-1 receptor agonists and incretin-based therapies	R: Established glycemic and weight benefits, with cardiovascular, kidney, and selected obesity-HFpEF benefits in studied populations.	Limited O/P: Direct human evidence for consistent effects on circulating FGF23 or soluble alpha-Klotho is insufficient; beta-Klotho findings should not be conflated with this axis.	S: Not established. No clinical evidence demonstrates biomarker mediation of treatment benefit.	Relevant as modification of obesity, glycemic burden, inflammation, and kidney risk; efficacy does not validate FGF23-Klotho biology.	Representative outcome evidence: (23) and related trials.
Finerenone and nonsteroidal MRAs	R: Established kidney and cardiovascular benefits in indicated T2D-CKD populations; HF benefit applies to studied HFmrEF/HFpEF populations and endpoints.	P/S: MR-FGF23 biological interaction is mechanistically plausible, but direct human pathway evidence is insufficient.	S: Not established. No mediation analysis has shown that FGF23 or soluble Klotho changes explain clinical benefit.	Clinical benefit supports MR antagonism, not FGF23-Klotho targeting.	Outcome trials: (21, 24, 37); mechanistic interaction remains hypothesis-generating.
Weight-loss strategies	R: Selected randomized studies support symptom, function, and weight improvement in obesity-related HFpEF; broader lifestyle evidence is supportive.	S or limited O/P: Direct effects on FGF23 or soluble Klotho are insufficiently characterized.	S: Not established.	Benefits validate treatment of obesity-related mechanisms but do not establish FGF23-Klotho involvement or mediation.	Representative evidence: (23, 48, 50, 51) as applicable.
Phosphate-lowering and CKD-MBD interventions	Guideline-established for progressively or persistently elevated serum phosphate and CKD-MBD management; R evidence has not established cardiovascular or HF benefit.	O/P: Phosphate burden and FGF23 are biologically linked, but binder trials have not consistently lowered FGF23 or demonstrated that FGF23 reduction improves clinical outcomes.	S: Not established. No mediation analysis has shown that changes in phosphate or FGF23 explain cardiovascular or HF outcomes.	Use for established hyperphosphatemia and CKD-MBD indications, not for cardiovascular prevention or treatment selection based solely on circulating FGF23.	Guideline: (39); randomized mineral-metabolism and phosphate-binder trials: (101–104).
Direct FGF23-, FGFR4-, or Klotho-directed strategies	No R evidence: No established HF outcome benefit and no validated treatment-selection threshold.	P: Evidence is predominantly preclinical or translational; direct suppression, selective FGFR4 blockade, soluble Klotho administration, and Klotho restoration remain investigational.	S: Human therapeutic efficacy, treatment-response mediation, and biomarker-guided selection remain unproven.	Investigational only. Broad FGF23 suppression may be hazardous; pathway-directed strategies require prospective safety and outcome evaluation.	Preclinical/translational evidence: (15, 19, 55, 56, 61, 66) as applicable.

Evidence labels: R, randomized clinical evidence; O, human observational or translational evidence; P, preclinical mechanistic evidence; S, speculative or hypothesis-generating inference. Combined labels indicate that different components of the same pathway are supported by different evidence types. Labels denote study type and clinical directness rather than effect size or formal certainty; detailed risk-of-bias and certainty assessments are provided in **Supplementary Tables 2, S3**. Randomized clinical efficacy of a parent therapy does not confer an R label on an untested FGF23-Klotho mechanism. Direct pathway engagement and mediation must be evaluated separately with therapy-specific prospective data.

CKD, chronic kidney disease; FGF23, fibroblast growth factor 23; FGFR4, fibroblast growth factor receptor 4; GLP-1, glucagon-like peptide-1; HF, heart failure; HFpEF, heart failure with preserved ejection fraction; HFmrEF, heart failure with mildly reduced ejection fraction; MRA, mineralocorticoid receptor antagonist; SGLT2, sodium-glucose cotransporter 2; T2D, type 2 diabetes.

## SGLT2 inhibitors

Established clinical evidence [R]. Large randomized trials, including DAPA-CKD, EMPA-KIDNEY, EMPEROR-Preserved, and DELIVER, have established kidney and heart-failure benefits of SGLT2 inhibitors in the populations studied (20, 22, 25, 38). These findings support their use according to established clinical indications. Additional randomized outcome trials and a trial-level meta-analysis across HFrEF, type 2 diabetes, CKD, and recent worsening heart failure broaden this evidence base (78–86).

Direct FGF23-Klotho evidence [limited O/P; mediation S]. SGLT2 inhibition may alter kidney function, tubular phosphate handling, and the broader mineral-metabolism environment in which circulating FGF23 and soluble Klotho are measured. However, therapy-specific effects on intact versus C-terminal FGF23 and soluble Klotho remain insufficiently characterized, and no clinical mediation evidence has established that changes in these biomarkers account for improved kidney or heart-failure outcomes. SGLT2 inhibitors should therefore be interpreted as established cardiorenal therapies rather than validated FGF23-Klotho-directed interventions.

## GLP-1 receptor agonists and incretin-based therapies

Established clinical evidence [R]. GLP-1 receptor agonists and related incretin-based therapies address both the adipose-metabolic and glycemic-control components of CKM-HF. In addition to improving glycemic control and body weight, randomized evidence supports cardiovascular, kidney-protective, weight-management, and selected obesity-related HFpEF benefits in the populations studied (23, 36, 44–46, 50, 51, 87–95).

Direct FGF23-Klotho evidence [limited O/P; mediation S]. Direct human evidence showing consistent modification of circulating FGF23 or soluble α-Klotho remains insufficient, and no outcome trial has established biomarker mediation of clinical benefit.

## RAAS blockade and mineralocorticoid receptor antagonism

Established clinical evidence [R]. RAAS blockade and mineralocorticoid receptor antagonism have established kidney, cardiovascular, and heart-failure benefits when used according to guideline-supported and trial-defined indications. For finerenone, FIDELIO-DKD and FIGARO-DKD support kidney and cardiovascular benefits in patients with type 2 diabetes and CKD. Earlier randomized trials of spironolactone and angiotensin-neprilysin inhibition in HFpEF provide additional context for neurohormonal and RAAS-related treatment strategies (96, 97). FINEARTS-HF, published online on September 1, 2024, subsequently extended randomized clinical evidence for finerenone to patients with heart failure and mildly reduced or preserved ejection fraction (21, 24, 37, 98). Importantly, FINEARTS-HF was published after the AHA CKM Presidential Advisory of October 9, 2023; its findings should therefore be interpreted as subsequent evidence that complements and updates the CKM treatment landscape rather than as evidence incorporated into the original AHA advisory.

Direct FGF23-Klotho evidence [P/S]. Biological interactions among mineralocorticoid receptor activation, inflammation, fibrosis, phosphate metabolism, and FGF23 signaling are mechanistically plausible, but direct human evidence that RAAS blockade or finerenone modifies FGF23-Klotho signaling remains insufficient.

Evidence of mediation [S]. No clinical mediation analysis has demonstrated that treatment-related changes in FGF23 or soluble Klotho account for the kidney, cardiovascular, or heart-failure benefits of these therapies.

RAAS blockade and finerenone should therefore be used according to established clinical indications rather than on the basis of FGF23 or soluble Klotho measurements. Their clinical efficacy supports RAAS/MR-directed treatment but should not be interpreted as evidence of FGF23-Klotho pathway engagement or mediation. Clinical interpretation and monitoring should also account for obesity-related suppression of natriuretic peptides and use standardized echocardiographic chamber quantification (99, 100).

## Combination therapy: guideline-informed sequencing, monitoring, and multidisciplinary referral

Established therapeutic indications [guideline-supported/R]; sequencing framework [not prospectively validated]. Combination therapy should be prioritized according to the dominant clinical problem, established treatment indications, kidney function, potassium concentration, volume status, glycemic and obesity burden, tolerability, and patient preferences rather than according to circulating FGF23 or soluble Klotho concentrations. In patients with active congestion, decongestion and clinical stabilization take priority. In stable patients with heart failure or eligible CKD, an SGLT2 inhibitor may be introduced early because of its established heart-failure and kidney benefits. ACE inhibitor or angiotensin-receptor blocker therapy should be prioritized in patients with hypertension and albuminuric CKD and titrated to the highest tolerated guideline-supported dose. In patients with type 2 diabetes and persistent albuminuria despite maximally tolerated RAAS blockade, finerenone may be added when kidney function and serum potassium meet guideline and regulatory criteria. GLP-1 receptor agonist-based therapy may be considered when obesity, inadequate glycemic control, or an adipose-metabolic HFpEF pattern represents a major treatment target (2, 20–25, 28–31, 37, 50).

These therapies are complementary rather than mutually exclusive, but their initiation and escalation should be individualized. Blood pressure, serum creatinine/eGFR, serum potassium, body weight, congestion, orthostatic symptoms, diuretic exposure, glycemia, and treatment tolerability should be documented before and after treatment changes. **Table 3** summarizes a pragmatic framework for treatment prioritization, monitoring, and multidisciplinary referral. The proposed sequence is guideline-informed and intended to support clinical reasoning; it has not been prospectively validated as a universal treatment algorithm.

**Table 3:** Guideline-informed prioritization, monitoring, and specialty-referral framework for established therapies in CKM-related heart failure.

Dominant clinical context	Priority treatment consideration	Subsequent combination strategy	Core monitoring	Specialty referral or coordination
Active congestion or recent HF decompensation	Prioritize clinical stabilization and loop-diuretic-based decongestion; reassess perfusion, blood pressure, kidney function, and precipitating factors. Introduce phenotype-appropriate HF therapy once stable (2, 4, 5).	After euvolemia or clear improvement, layer organ-protective therapies according to HF, CKD, diabetes, albuminuria, and obesity indications. Avoid simultaneous rapid escalation when hypotension, acute kidney injury, or marked volume depletion is present.	Symptoms and signs of congestion; daily or serial body weight when clinically relevant; orthostatic blood pressure; serum creatinine/eGFR, sodium, and potassium; diuretic response and adverse effects.	Cardiology: uncertain HF diagnosis/phenotype, recurrent hospitalization, refractory congestion, significant arrhythmia, valvular disease, pulmonary hypertension, or advanced-HF features. Nephrology: persistent or unexplained kidney-function deterioration during decongestion.
Stable HF and/or CKD with an established indication for SGLT2 inhibition	Consider early SGLT2 inhibitor therapy in eligible HF or CKD populations, independent of FGF23 or soluble Klotho measurements (20, 22, 25, 38).	Combine with RAAS blockade, finerenone, and metabolic/weight-directed therapy when each is independently indicated and tolerated. Review diuretic dose in patients at risk of volume depletion.	Baseline eGFR, blood pressure, volume status, body weight, and glycemia. Reassess earlier when frailty, low blood pressure, high diuretic burden, or dehydration risk is present. A modest reversible early eGFR decline is generally not, by itself, an indication to discontinue therapy.	Cardiology or nephrology: symptomatic hypotension, persistent volume depletion, unexpected sustained eGFR decline, recurrent acute kidney injury, or difficulty balancing decongestion with kidney protection.
Hypertension with albuminuric CKD or DKD	Prioritize an ACE inhibitor or ARB and titrate to the highest tolerated guideline-supported dose. Avoid dual ACE inhibitor-ARB therapy (28–31).	Add an SGLT2 inhibitor when indicated. Consider finerenone for persistent albuminuria in eligible patients with type 2 diabetes, adequate kidney function, and acceptable serum potassium.	Blood pressure, serum creatinine/eGFR, and potassium at baseline and within 2–4 weeks after RAAS inhibitor initiation or dose escalation; reassess albuminuria longitudinally.	Nephrology: rapid or unexplained eGFR decline, severe/persistent albuminuria, resistant hypertension, persistent potassium abnormality, eGFR <30 mL/min/1.73 m² or elevated kidney-failure risk, anemia, acidosis, or CKD-mineral and bone disorder.
Type 2 diabetes with persistent albuminuria despite tolerated RAAS blockade	Consider finerenone when guideline eligibility and local regulatory criteria are met; treatment eligibility should not be determined by FGF23 or soluble Klotho (21, 24, 37, 98).	Finerenone may be layered with RAAS blockade and an SGLT2 inhibitor when separately indicated and tolerated. Review concomitant drugs and diet that increase hyperkalemia risk.	Serum potassium and creatinine/eGFR at baseline; potassium approximately 1 month after initiation or dose adjustment and periodically thereafter (commonly every 4 months in stable patients), with earlier reassessment in higher-risk patients.	Nephrology: recurrent hyperkalemia, substantial kidney-function decline, advanced CKD, complex CKD complications, or inability to maintain indicated RAAS/MR therapy safely.
Obesity-related HFpEF, inadequately controlled type 2 diabetes, or dominant adipose-metabolic burden	Consider GLP-1 receptor agonist-based therapy according to established obesity, diabetes, cardiovascular, and kidney indications (23, 36, 50, 51).	Combine with SGLT2 inhibition and kidney-protective therapy when independently indicated. Adjust concomitant glucose-lowering treatment to reduce hypoglycemia risk and pair weight loss with nutrition and resistance/functional exercise when feasible.	Body weight, glycemia, gastrointestinal tolerance, hydration, nutritional intake, lean-mass preservation, kidney function when clinically indicated, and treatment adherence.	Endocrinology: complex glycemic regimens, recurrent hypo- or hyperglycemia, severe obesity requiring pharmacological escalation, uncertainty regarding incretin selection/titration, or major endocrine comorbidity. Cardiology: persistent HF symptoms despite metabolic improvement.
Multisystem CKM-HF, competing indications, or diagnostic uncertainty	Address the most immediate clinical threat first (congestion, rapid CKD progression, severe hyperglycemia, symptomatic hypotension, hyperkalemia, or treatment toxicity), then construct a coordinated long-term plan.	Use staged multidisciplinary escalation rather than a biomarker-intensive universal sequence. FGF23 and soluble Klotho remain research biomarkers and should not direct therapy selection or sequencing.	Integrated review of HF symptoms, eGFR trajectory, UACR, potassium, blood pressure, glycemia, body weight, volume status, anemia, mineral parameters, adverse effects, adherence, cost, and access.	Joint cardiology-nephrology-endocrinology management when treatment in one domain destabilizes another, indications compete, adverse effects prevent evidence-based therapy, or the dominant driver of symptoms remains uncertain.

Therapy-specific efficacy is supported by randomized or guideline-established evidence; the proposed prioritization, sequencing, monitoring, and referral framework is guideline-informed and has not been prospectively validated as a universal treatment algorithm. It should not replace disease-specific guidelines, regulatory labeling, local protocols, individualized clinical judgment, or shared decision-making. Treatment selection and sequencing should be based on established HF, CKD, diabetes, obesity, blood-pressure, and albuminuria indications rather than FGF23 or soluble Klotho measurements. Local recommendations may differ regarding eGFR eligibility, potassium thresholds, dose adjustment, temporary interruption, and monitoring frequency. Treatment prioritization and sequencing should incorporate patient preferences, treatment burden, affordability and access, health literacy, cultural feasibility, caregiver support, and the patient’s ability and willingness to complete the proposed monitoring plan through shared decision-making.

Evidence status. Therapy-specific efficacy: R or guideline-established; proposed sequencing and referral framework: guideline-informed/S. Representative sources correspond to the manuscript references shown in the table.

ACE inhibitor, angiotensin-converting enzyme inhibitor; ARB, angiotensin receptor blocker; CKD, chronic kidney disease; CKM, cardiovascular-kidney-metabolic; DKD, diabetic kidney disease; eGFR, estimated glomerular filtration rate; FGF23, fibroblast growth factor 23; HF, heart failure; HFpEF, heart failure with preserved ejection fraction; MR, mineralocorticoid receptor; RAAS, renin-angiotensin-aldosterone system; SGLT2, sodium-glucose cotransporter 2; T2D, type 2 diabetes; UACR, urine albumin-creatinine ratio.

For FGF23-Klotho research, combination therapy remains an important source of time-varying confounding. SGLT2 inhibition, RAAS blockade, finerenone, and incretin-based treatment may alter kidney function, albuminuria, volume status, inflammation, body weight, phosphate handling, and circulating biomarker concentrations. FGF23 and soluble Klotho should therefore be interpreted longitudinally with careful documentation of medication initiation, dose changes, kidney function, mineral parameters, and volume status. A treatment-associated change in either biomarker should not be interpreted as direct pathway engagement or mediation of clinical benefit.

## Patient perspective and shared decision-making

Guideline-supported patient-centered practice. CKM-HF management frequently combines lifestyle modification, symptom self-management, and multiple therapies that differ in expected benefit, route of administration, adverse effects, monitoring requirements, and cost. Shared decision-making should therefore begin by identifying the outcomes that matter most to the individual patient, such as symptom relief, preservation of functional capacity and independence, avoidance of hospitalization, slowing of kidney-disease progression, improvement in glycemic or weight control, or minimization of treatment burden. The expected benefits, uncertainties, and monitoring requirements of each intervention should be communicated in accessible language rather than presented as a fixed universal treatment sequence.

Treatment selection and sequencing should also account for frailty, cognitive and functional status, health literacy, caregiver support, cultural and dietary feasibility, medication affordability and access, previous adverse effects, and willingness or ability to undergo repeated laboratory and clinical monitoring. When several therapies are indicated, a staged plan with agreed priorities, follow-up intervals, safety parameters, and opportunities to reconsider treatment may be more acceptable and feasible than simultaneous rapid escalation. Patient-reported symptoms, functional capacity, quality of life, adverse effects, and treatment burden should be assessed alongside eGFR, potassium, albuminuria, glycemia, body weight, congestion, and clinical events. Investigational FGF23 or soluble Klotho testing should not add cost or complexity to routine care outside research protocols because neither biomarker currently has validated clinical thresholds or demonstrated treatment-selection utility (1, 2, 4, 5, 28).

## Direct and investigational FGF23-Klotho-targeted strategies

Direct pathway-directed strategies [P/S; no established human efficacy]. Direct FGF23-, FGFR4-, or Klotho-directed treatment remains investigational. Within the evidence base reviewed, no randomized clinical outcome trial has established that direct suppression of FGF23, selective FGFR4 blockade, soluble Klotho administration, or restoration of endogenous Klotho improves heart-failure outcomes. No validated biomarker threshold currently identifies patients who should receive such an intervention. Other candidate approaches include vitamin D and PTH modulation, anti-inflammatory treatment, Klotho restoration, and selective interruption of pathological FGFR4-related cardiac signaling. However, vitamin D analogues may increase circulating FGF23, broad FGF23 neutralization may aggravate phosphate retention and adverse outcomes, and neither Klotho restoration nor FGFR4 inhibition has demonstrated clinical heart-failure benefit in humans (15, 19, 55, 56, 61, 66).

Phosphate management and cardiovascular-outcome evidence [guideline-supported management; cardiovascular benefit unproven]. Phosphate management in CKD should be individualized according to serial serum phosphate, calcium, PTH, kidney function, dietary exposure, and the broader CKD-mineral and bone disorder context rather than initiated solely because circulating FGF23 is elevated. Dietary counseling may prioritize reducing highly absorbable inorganic phosphate additives while maintaining adequate protein intake and overall nutritional adequacy. Current CKD-MBD guidance supports dietary phosphate limitation and phosphate-lowering treatment for progressively or persistently elevated serum phosphate, but does not support indiscriminate phosphate-binder treatment in normophosphatemic earlier-stage CKD. Evidence that dietary phosphate restriction itself reduces cardiovascular or heart-failure events remains insufficient (39).

Randomized clinical evidence [R; cardiovascular benefit unproven]. Randomized intervention studies have not established that modification of phosphate or related mineral-metabolism pathways consistently improves cardiovascular outcomes. EVOLVE was not a phosphate-binder trial, but evaluated cinacalcet in patients undergoing dialysis with moderate-to-severe secondary hyperparathyroidism. In the unadjusted intention-to-treat analysis, cinacalcet did not significantly reduce the primary composite of death or major cardiovascular events, illustrating that modification of mineral-metabolism abnormalities does not necessarily translate into cardiovascular benefit (101). In patients with moderate CKD and normal or near-normal serum phosphate, Block et al. found that phosphate-binder therapy produced only a modest reduction in serum phosphate and was associated with greater progression of coronary-artery and abdominal-aortic calcification than placebo, with the calcification signal most evident for calcium-containing binders (102). In IMPROVE-CKD, treatment with lanthanum carbonate for 96 weeks did not improve arterial stiffness or aortic calcification compared with placebo in patients with stage 3b–4 CKD (103). In the LANDMARK trial, lanthanum carbonate was not superior to calcium carbonate in reducing composite cardiovascular events among patients with hyperphosphatemia undergoing hemodialysis (104).

Taken together, these findings indicate that phosphate binders remain appropriate for established hyperphosphatemia and CKD-MBD management according to guideline-supported indications, but should not be presented as validated therapies for preventing heart failure or cardiovascular events. They should also not be initiated solely to lower circulating FGF23 or to reduce presumed FGF23-related cardiac risk (39, 101–104).

Research and trial-design implications [S]. The appropriate near-term role of the FGF23-Klotho axis is hypothesis generation and mechanism-based trial design. Prospective studies may use FGF23 or soluble Klotho to enrich mechanistic substudies, provided that assay methods, kidney function, phosphate balance, treatment exposure, mineral parameters, and safety outcomes are prespecified. Such use should be clearly distinguished from routine clinical risk stratification, treatment selection, or therapeutic monitoring.

## Biomarkers in CKM-related heart failure: current clinical practice and research applications

Established clinical assessment [guideline-established]. Biomarker assessment in CKM-related heart failure should distinguish markers that are established for routine clinical care from biomarkers that remain investigational. Current clinical practice should continue to rely on standardized and actionable measures, including eGFR, urine albumin-creatinine ratio, blood pressure, HbA1c, body mass index or waist circumference, potassium, natriuretic peptides, and guideline-supported cardiac imaging. Phosphate, calcium, PTH, and vitamin D status may also be assessed when clinically indicated by CKD-mineral and bone disorder or other established clinical contexts (3, 7, 8, 26–32, 39, 40).

Established markers provide complementary but clinically distinct information. eGFR and albuminuria quantify kidney filtration and kidney injury; phosphate, calcium, PTH, and vitamin D characterize established aspects of mineral metabolism; natriuretic peptides primarily reflect myocardial wall stress and congestion; and cardiac imaging defines structural and functional abnormalities, including LVH, fibrosis, diastolic dysfunction, and elevated filling pressures. These measures therefore remain the foundation of current CKM-HF assessment.

Investigational biomarker evidence [O/P; clinical utility S]. Circulating FGF23 and soluble Klotho occupy a different evidentiary category. Although observational studies have associated higher FGF23 with LVH, heart-failure risk, mortality, and kidney progression, neither FGF23 nor soluble Klotho currently has sufficiently standardized assays, validated reference intervals, prospectively confirmed decision thresholds, or demonstrated outcome benefit from biomarker-guided management. They should therefore not be presented as routine diagnostic, prognostic, treatment-selection, or treatment-response tools.

FGF23 and soluble Klotho have been proposed to provide different, but as yet unvalidated, biological information. FGF23 may reflect a broader state of mineral-endocrine stress involving phosphate handling, nephron loss, inflammation, and adaptive or maladaptive FGF receptor signaling, whereas soluble Klotho may reflect loss of renal endocrine reserve and protective signaling that is not directly measured by eGFR or albuminuria. In theory, these biomarkers could help investigate why patients with similar kidney function or phosphate concentrations show different patterns of cardiac remodeling. However, this proposed information is mechanistic and hypothesis-generating rather than clinically established.

Importantly, neither FGF23 nor soluble Klotho has been shown to provide consistent incremental discrimination, calibration, risk reclassification, decision-curve benefit, or treatment-selection value beyond models containing eGFR, albuminuria, phosphate, PTH, natriuretic peptides, and cardiac imaging. Because circulating FGF23 is strongly influenced by kidney function, phosphate balance, PTH, vitamin D status, inflammation, anemia, and treatment exposure, part of its association with adverse outcomes may overlap with information already captured by established markers. Soluble Klotho interpretation is further limited by assay heterogeneity. Accordingly, statistical association alone should not be interpreted as evidence of incremental clinical utility.

**Figure 5:**
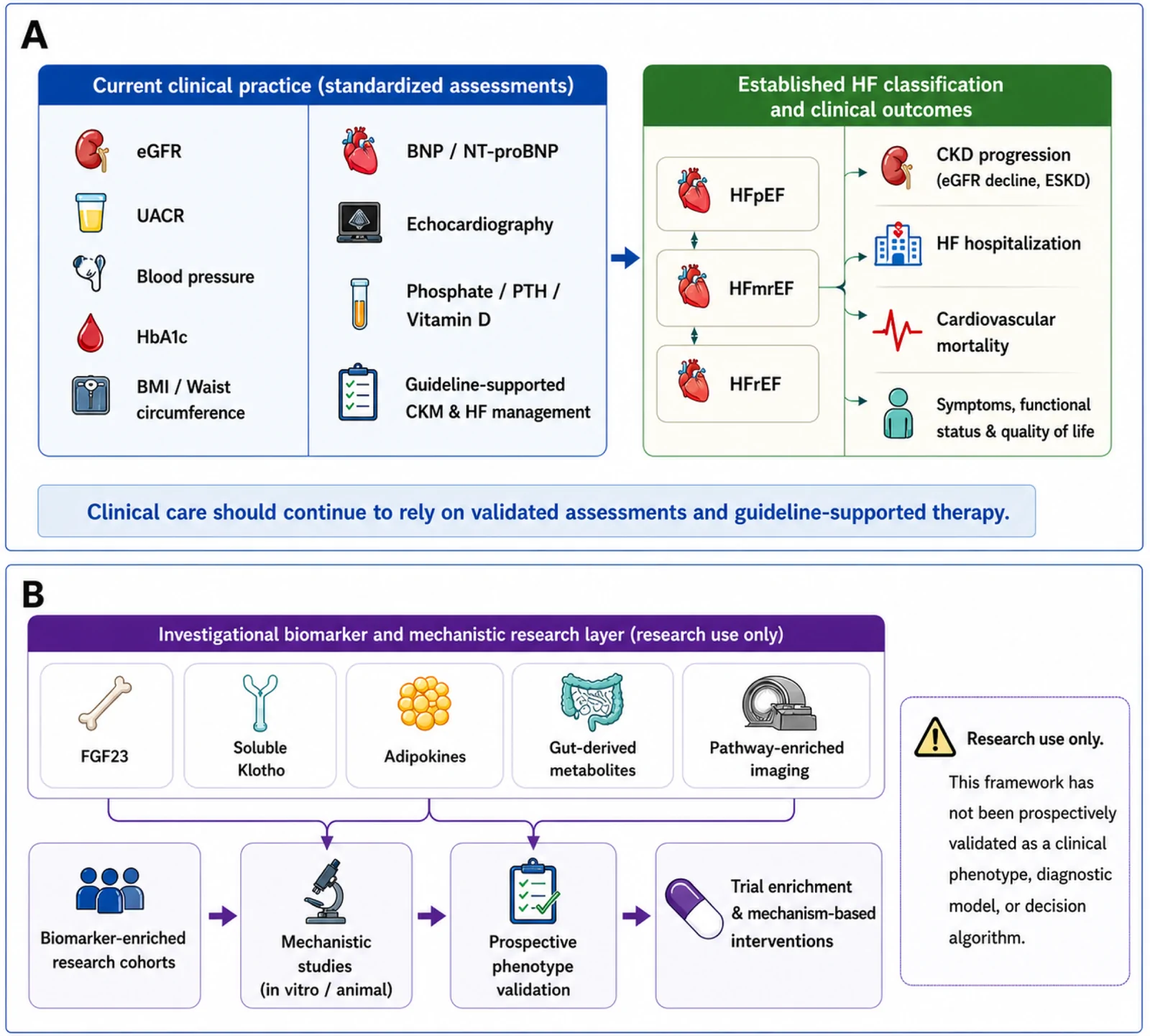
Established clinical assessment and investigational biomarker research in CKM-related heart failure. **(A)** Current clinical practice relies on standardized kidney, metabolic, cardiovascular, mineral, and cardiac assessments together with guideline-supported treatment. Mineral parameters, including phosphate, PTH, and vitamin D status, are assessed when clinically indicated. Established heart-failure classification and clinical outcomes include HFpEF, HFmrEF, HFrEF, CKD progression, heart-failure hospitalization, cardiovascular mortality, symptoms, functional status, and quality of life. **(B)** The investigational research layer includes FGF23, soluble Klotho, adipokines, gut-derived metabolites, pathway-enriched imaging, biomarker-enriched cohorts, mechanistic studies, prospective phenotype validation, and trial enrichment. These components are intended for hypothesis generation and prospective research rather than routine clinical decision-making. The proposed mineral-endocrine-enriched phenotype has not been prospectively validated and currently lacks consensus diagnostic criteria, standardized biomarker thresholds, weighting rules, and a validated classification algorithm. This framework should not be used for routine screening, diagnosis, prognosis, treatment selection, treatment intensification, or response monitoring.

**Table 4** and **Figure 5A** summarize established clinical assessment and management in CKM-related heart failure, whereas **Figure 5B** presents a separate investigational framework for biomarker validation, research phenotyping, and trial enrichment. Current clinical care should continue to rely on validated and clinically actionable measures, including eGFR, albuminuria, mineral parameters when clinically indicated, natriuretic peptides, cardiac imaging, and guideline-supported therapy. Circulating FGF23 and soluble Klotho may be evaluated in mechanistic studies, prospective phenotype-validation cohorts, and biomarker-enriched trials, but neither biomarker has validated clinical thresholds or demonstrated incremental utility beyond established kidney, mineral, cardiac, and imaging markers. Accordingly, neither should independently trigger diagnosis, additional imaging, treatment initiation or intensification, treatment withholding, or response monitoring. The proposed mineral-endocrine-enriched phenotype remains a hypothesis-generating research construct and lacks consensus diagnostic criteria, validated biomarker cutoffs, weighting rules, and prospective validation. **Figure 5B** is therefore intended for research and clinical reasoning and has not been prospectively validated as a clinical decision algorithm (15, 16, 66).

**Table 4:** Established clinical markers and investigational biomarkers in CKM-related heart failure: evidence type and clinical status.

Biomarker category	Marker	Established clinical use	Potential incremental information and investigational use	Major limitations	Evidence type and current status
Kidney function assessment	eGFR	CKD staging, kidney-risk assessment, and treatment monitoring	Reference framework for CKM phenotyping and interpretation of emerging biomarkers	Affected by acute kidney injury, age, muscle mass, and comorbidity	O; guideline-established clinical use
Kidney injury assessment	Urine albumin-creatinine ratio (UACR)	Albuminuria classification, CKD prognosis, and therapeutic decision-making	Identification of high-risk CKM patterns and reference adjustment in biomarker studies	Biological variability and influence of acute conditions	O; guideline-established clinical use
Cardiac injury and HF assessment	BNP/NT-proBNP; echocardiographic and selected CMR parameters	HF diagnosis, phenotyping, prognosis, and monitoring	Integration with candidate multimarker research models	Influenced by obesity, atrial fibrillation, renal dysfunction, loading conditions, and referral context	O; guideline-established clinical use
Metabolic and cardiovascular risk assessment	HbA1c; blood pressure; BMI/waist circumference; lipid profile	Assessment of diabetes, obesity, and cardiovascular risk burden	Characterization of CKM metabolic patterns	Limited ability to directly capture mineral-endocrine alterations	O; guideline-established clinical use
Mineral metabolism assessment	Phosphate, calcium, PTH, 25(OH)D	CKD-mineral and bone disorder evaluation and clinically indicated management	Contextual interpretation of FGF23-Klotho biology	Requires interpretation within kidney function, treatment exposure, and mineral-metabolism context	O; guideline-established in appropriate CKD-MBD contexts
FGF23-Klotho axis biomarkers	Intact or C-terminal FGF23; soluble Klotho	No established routine role in HF diagnosis, prognosis, treatment selection, or monitoring	FGF23 may reflect candidate mineral-endocrine stress; soluble Klotho may reflect candidate loss of renal endocrine reserve. Potential uses are mechanistic phenotyping and biomarker-enriched research.	FGF23 assay non-equivalence; limited soluble Klotho standardization; no validated thresholds; no demonstrated improvement in discrimination, reclassification, decision-making, or outcomes beyond established markers	O/P; clinical utility, thresholds, and biomarker-guided care remain S
Gut-derived metabolic biomarkers	TMAO and related gut-derived metabolites	No established application in CKM-HF management	Research into inflammation, metabolism, renal clearance, and cardiovascular-risk pathways	Strong influence from diet, microbiota composition, kidney clearance, and medications	O/P; routine clinical use and treatment relevance remain S
Adipose-derived biomarkers	Adipokines, including leptin and adiponectin	No established routine clinical role	Research into obesity-related HF patterns and metabolic signaling	Assay variability, phenotype overlap, and uncertain incremental clinical value	O/P; incremental clinical utility remains S

Evidence labels: R, randomized clinical evidence; O, human observational or translational evidence; P, preclinical mechanistic evidence; S, speculative or hypothesis-generating inference. Combined labels indicate that different components of the same pathway are supported by different evidence types. Labels denote study type and clinical directness rather than effect size or formal certainty; detailed risk-of-bias and certainty assessments are provided in **Supplementary Tables 2, S3**. This framework is intended for research and clinical reasoning and has not been prospectively validated as a clinical decision algorithm. No randomized biomarker-guided evidence has demonstrated that measuring FGF23 or soluble Klotho improves diagnosis, prognosis, treatment selection, response monitoring, or patient outcomes.

BNP, B-type natriuretic peptide; CKD-MBD, chronic kidney disease-mineral and bone disorder; CMR, cardiac magnetic resonance; eGFR, estimated glomerular filtration rate; FGF23, fibroblast growth factor 23; HF, heart failure; PTH, parathyroid hormone; TMAO, trimethylamine N-oxide; UACR, urine albumin-creatinine ratio.

## Discussion: controversies, knowledge gaps, and future directions

Several high-priority questions remain unresolved before endocrine crosstalk can be translated into routine CKM-HF phenotyping or intervention design. These questions concern causality, assay standardization, phenotype specificity, treatment integration, and implementation across real-world care settings. Framing these issues explicitly is important because the translational bottleneck in this field is no longer the absence of plausible mechanisms, but the difficulty of deciding which mechanisms are sufficiently reproducible, measurable, and actionable to change care.

Causality remains the first and most fundamental challenge. Animal and cellular studies support the biological plausibility that FGF23 may promote hypertrophic signaling through FGFR4-related pathways under selected experimental conditions, whereas human studies predominantly demonstrate associations between higher circulating FGF23 and LVH, incident heart failure, heart-failure subtype, cardiovascular events, mortality, and kidney progression (17, 18, 33–35, 57–61). These two evidence streams are not equivalent: experimental mechanisms cannot be assumed to operate with the same magnitude or clinical relevance in humans, and observational associations cannot distinguish whether FGF23 is a causal mediator, a compensatory endocrine response, or a marker of CKD severity and mineral stress. Residual confounding by eGFR, phosphate balance, inflammation, anemia, frailty, and medication exposure, together with assay heterogeneity and possible reverse causation, further limits causal interpretation. Direct human causality would require longitudinal evidence showing that changes in FGF23 precede cardiac remodeling, robust mediation or causal-inference analyses, and ultimately interventional evidence demonstrating that selective pathway modification improves cardiac outcomes without worsening phosphate balance or vascular safety. Such evidence is currently unavailable. **Table 5** summarizes the major controversies and research priorities arising from these limitations. Rather than weakening the CKM framework, the distinction between mechanistic plausibility, observational association, and demonstrated causality provides a roadmap for the next generation of studies. A second challenge concerns assay standardization. FGF23 assays measure intact or C-terminal fragments, and results may not be interchangeable. Soluble Klotho assays are even more heterogeneous (15, 16, 66). Without assay standardization, reference intervals, and clinically meaningful thresholds, precision medicine will remain difficult. A third unresolved issue is phenotype specificity. HFpEF is not one disease. Obesity-related HFpEF, CKD-related HFpEF, atrial fibrillation-dominant HFpEF, pulmonary hypertension-associated HFpEF, and infiltrative or high-output mimics may have different endocrine signatures (3, 6–14, 48, 49). FGF23 may be most informative in kidney-mineral phenotypes, whereas adipokines and inflammatory markers may dominate obesity-HFpEF. Future trials should avoid treating HFpEF as a single category and should include CKM biomarker substudies with EF-stratified adjudication (33–35, 51). Sex- and gender-specific considerations [O/S]. Biological sex and gender-related factors should be considered separately because they may influence CKM-HF through different mechanisms. Women constitute a larger proportion of HFpEF populations and may differ from men in ventricular-vascular structure, adiposity distribution, exercise hemodynamics, symptom burden, and comorbidity patterns. Sex differences have also been reported in CKD progression, whereas circulating FGF23 distributions and their associations with cardiovascular outcomes may vary by sex. However, these findings remain heterogeneous and do not support sex-specific FGF23 or soluble Klotho thresholds. Available randomized subgroup analyses suggest that the clinical benefit of dapagliflozin is broadly consistent in women and men across the ejection-fraction spectrum, but sex-specific efficacy, safety, tolerability, and patient-reported outcomes remain insufficiently characterized for several other CKM-HF therapies and should be prospectively evaluated. Gender identity and gender-related social factors may additionally influence access to care, treatment burden, adherence, and shared decision-making. Future CKM-HF studies should therefore report sex-disaggregated biomarker and outcome data, prespecify treatment-by-sex interaction analyses, and, where feasible, record sex assigned at birth and gender identity separately rather than assuming that findings derived from binary sex categories generalize to transgender and gender-diverse populations (6, 10, 20–25, 33–37, 105–107). A fourth question is how endocrine phenotyping should be integrated with treatment selection. SGLT2 inhibitors, GLP-1 RAs, finerenone, RAAS blockade, and weight-loss strategies now overlap in patients with diabetes, CKD, obesity, and HFpEF (2, 20–25, 36–38, 50). The next step is to prospectively compare treatment sequence, combination strategies, and phenotype-specific benefit rather than simply restating individual drug efficacy. Pending such evidence, **Table 3** provides a guideline-informed clinical-reasoning framework that prioritizes established treatment indications, safety monitoring, and multidisciplinary referral without using FGF23 or soluble Klotho to direct therapy. Finally, implementation across real-world CKM-HF care remains uncertain. CKM-HF care requires coordination among cardiology, endocrinology, nephrology, primary care, nutrition, and rehabilitation. Biomarker panels and combination therapies will fail if they create complexity without actionable pathways. Future guidelines should define when to refer, which markers change management, and how to monitor therapy combinations while operationalizing the patient-centered and shared decision-making principles outlined above, including treatment burden, affordability, access, and monitoring feasibility. A staged care model involving primary care, endocrinology, nephrology, cardiology, nutrition, and rehabilitation may be more practical than a universal biomarker-intensive strategy for all patients (1, 6, 28). A further gap is equity and generalizability. CKM syndrome is shaped by diet quality, access to kidney and diabetes care, medication affordability, environmental stress, and structural determinants of cardiometabolic risk. Biomarker-based precision medicine could widen disparities if specialized assays are available only in referral centers or trials. Future studies should therefore report population characteristics, social risk, kidney function strata, access barriers, and the sex- and gender-related variables outlined above, and should test whether simplified CKM pathways can be implemented in routine care without losing the biological nuance that makes the framework useful (1).

**Table 5:** Key controversies and future research priorities in the FGF23-Klotho axis and CKM-related heart failure.

Controversy or research question	Current evidence type	Current interpretation	Key limitations	Priority research direction
Does FGF23 directly cause human cardiac remodeling?	P for direct mechanism; O for human association; causal inference remains S	FGFR4-related hypertrophic signaling supports biological plausibility, while human data show association rather than established causation.	Residual confounding, reverse causation, assay heterogeneity, and absence of selective human intervention evidence.	Longitudinal cohorts with repeated assays, causal-inference analyses, human mechanistic studies, and selective pathway-targeted trials with mineral safety monitoring.
Does a distinct FGF23-Klotho-associated HF phenotype exist?	O/P for component features; the proposed phenotype remains S	The candidate mineral-endocrine-enriched pattern is a hypothesis-generating construct, not a validated clinical phenotype or endotype.	No diagnostic criteria, biomarker thresholds, weighting rules, reproducibility data, or prospective external validation.	Prospective multicenter derivation using clustering or latent-class methods, followed by internal validation, external replication, temporal-stability testing, and treatment-interaction assessment.
Do FGF23 or soluble Klotho add value beyond established markers?	Limited O; incremental clinical utility remains S	Candidate mechanistic information may differ from individual conventional markers, but added diagnostic, prognostic, and decision value is unproven.	No consistent improvement in discrimination, calibration, reclassification, decision-curve net benefit, management, or outcomes.	Add biomarkers individually and jointly to prespecified reference models containing eGFR, albuminuria, mineral markers, natriuretic peptides, and imaging; externally validate clinical utility.
Do established cardiometabolic therapies act through FGF23-Klotho?	R for clinical efficacy; direct pathway evidence is limited O/P or P/S; mediation remains S	SGLT2 inhibitors, GLP-1 receptor agonists, and finerenone have proven clinical effects for studied indications, but pathway engagement and mediation are not established.	Outcome trials generally did not prespecify serial FGF23/Klotho measurement or formal mediation analysis.	Prospective mechanistic substudies with standardized assays, serial sampling, treatment-exposure documentation, and formal mediation analyses.
Can FGFR4 blockade or Klotho restoration improve HF outcomes?	P for mechanistic plausibility; human efficacy remains S	Direct pathway-directed treatment remains investigational, and broad FGF23 suppression may disrupt adaptive phosphate regulation.	Preclinical indirectness, safety concerns, no validated selection threshold, and no randomized HF outcome evidence.	Early-phase target-engagement and safety trials, selective rather than broad pathway modulation, and prespecified phosphate, calcium, PTH, vascular, kidney, and cardiac endpoints.
Are gut-derived metabolites direct modifiers of the FGF23-Klotho axis?	O/P for cardiovascular association; direct axis interaction and therapeutic relevance remain S	Gut metabolites are contextual modifiers and potential confounders rather than a coequal validated FGF23-Klotho pathway.	Diet, microbiota composition, hepatic metabolism, kidney clearance, medications, and heterogeneous assays.	Mechanistic studies integrating microbiome, metabolomics, kidney function, and mineral-endocrine measures, followed by carefully selected interventional studies.

Evidence labels: R, randomized clinical evidence; O, human observational or translational evidence; P, preclinical mechanistic evidence; S, speculative or hypothesis-generating inference. Combined labels indicate that different components of the same pathway are supported by different evidence types. Labels denote study type and clinical directness rather than effect size or formal certainty; detailed risk-of-bias and certainty assessments are provided in **Supplementary Tables 2, S3**. The **S** label is used for unvalidated phenotypes, causal claims, treatment mediation, or therapeutic implications that exceed the direct evidence base.

CKM, cardiovascular-kidney-metabolic; eGFR, estimated glomerular filtration rate; FGF23, fibroblast growth factor 23; FGFR4, fibroblast growth factor receptor 4; HF, heart failure; PTH, parathyroid hormone.

The therapeutic evidence requires particularly cautious interpretation. Randomized benefits of SGLT2 inhibitors, GLP-1 receptor agonist-based therapies, and finerenone validate these interventions for their studied clinical indications but do not validate FGF23 or soluble Klotho as biomarkers, mediators, or treatment targets. Changes in kidney function, albuminuria, volume status, body weight, inflammation, or phosphate handling may secondarily alter circulating FGF23 or Klotho without indicating direct pathway engagement. Within the evidence reviewed, formal mediation data linking treatment-induced changes in FGF23 or soluble Klotho to improved heart-failure or kidney outcomes are lacking.

Hypothesis-generating research priorities [S]. Future studies should prospectively derive and validate the proposed candidate mineral-endocrine-enriched heart-failure phenotype rather than assume that it already represents a distinct clinical entity or use it immediately as a trial-enrichment criterion. Prospective multicenter cohorts should prespecify whether intact or C-terminal FGF23 is measured, report assay platforms and sampling conditions, and use harmonized soluble Klotho assays where feasible (15, 16, 66). Candidate biomarker thresholds should be derived rather than adopted retrospectively. Standardized data should also be collected for eGFR, albuminuria, phosphate, calcium, PTH, vitamin D status, hemoglobin and iron status, inflammatory markers, medication exposure, volume status, congestion, cardiac structure and function, and established HF phenotype (6, 18, 28, 33–35, 39, 57–60, 64–66). These variables are necessary to distinguish a potential FGF23-Klotho-associated pattern from the effects of kidney dysfunction, phosphate burden, anemia, obesity, diabetes, inflammation, and treatment exposure. Phenotype derivation should use prespecified statistical approaches, including data-driven clustering or latent-class analysis, followed by internal validation and external replication in independent populations. Subsequent studies should assess classification reproducibility, temporal stability, incremental prognostic value beyond established CKM and HF models, treatment interactions, and clinical utility. Only after reproducible external validation, definition of clinically interpretable thresholds, and demonstration of incremental value should the proposed phenotype be considered for biomarker-enriched interventional trials. Such trials should then separate mechanistic endpoints—including FGF23, soluble Klotho, phosphate handling, LV mass, fibrosis, and diastolic function—from clinical outcomes such as symptoms, HF hospitalization, kidney progression, and mortality, while prespecifying mineral-metabolism safety monitoring (17, 19, 39, 55, 56, 61). Implementation considerations should remain secondary to prospective validation. Current CKM-HF care should continue to rely on established clinical variables, including diabetes, obesity, blood pressure, eGFR, albuminuria, congestion, cardiac imaging, and guideline-supported therapy (1, 2, 28, 40). Multidisciplinary coordination among cardiology, nephrology, endocrinology, primary care, nutrition, and rehabilitation remains important for delivering this established care. By contrast, FGF23, soluble Klotho, and the proposed mineral-endocrine-enriched phenotype should remain within research protocols until standardized measurements, reproducible classification criteria, incremental clinical value, and actionable management consequences have been demonstrated. Future implementation studies should therefore evaluate feasibility, cost, access, patient preferences, and whether adding these investigational measures improves care beyond established CKM and HF assessments.

Future studies should evaluate incremental value explicitly rather than infer it from independent statistical associations. A prespecified reference model should include established variables such as age, sex, eGFR, albuminuria, phosphate, calcium, PTH, vitamin D status, hemoglobin, diabetes, blood pressure, natriuretic peptides, and cardiac imaging findings. FGF23 and soluble Klotho should then be added individually and jointly to this reference model. Studies should assess changes in discrimination, calibration, risk reclassification, decision-curve net benefit, and clinically relevant outcomes, followed by external validation in independent cohorts. Incremental prognostic performance alone would still be insufficient for routine use unless biomarker measurement also changes management or improves patient outcomes.

## Limitations of this review

This review has several limitations. First, it is a structured narrative review rather than a systematic review or meta-analysis; therefore, it was not conducted under a prospectively registered protocol, and no quantitative synthesis was undertaken. The original search and screening log was not prospectively preserved and was reconstructed by rerunning the database searches during revision. Although the revised searches and eligibility decisions were documented, this *post hoc* reconstruction may still have introduced selection bias. Second, although DS and XW independently performed design-specific methodological appraisal and a proposition-level GRADE-informed certainty assessment, the use of different instruments across heterogeneous study designs inevitably required judgment, and the resulting assessments cannot be meaningfully reduced to a single cross-design numerical score. Third, several clinically attractive areas—particularly FGF23-Klotho biology, soluble Klotho measurement, and gut-metabolite phenotyping—remain limited by assay heterogeneity, phenotype overlap, residual confounding, and incomplete interventional validation. Fourth, because CKM-related heart failure spans multiple specialties, the evidence base is stronger for some renal and metabolic outcomes than for heart-failure subtype-specific outcomes.

Clinical translation also remains substantially limited. FGF23 assays quantify biologically distinct intact or C-terminal forms and are not necessarily interchangeable, whereas soluble Klotho assays remain heterogeneous and lack harmonized reference intervals. More importantly, neither circulating FGF23 nor soluble Klotho has a validated CKM-HF decision threshold, demonstrated incremental clinical utility beyond established markers, or evidence that biomarker-guided management improves patient outcomes. The proposed FGF23-Klotho-associated heart-failure phenotype is similarly unvalidated. It has not been derived using data-driven clustering, tested against prespecified diagnostic criteria, externally replicated, or prospectively evaluated for reproducibility, prognosis, treatment interaction, or incremental value beyond established CKM and heart-failure classifications. No biomarker cutoff, required combination of kidney and cardiac findings, or classification threshold can currently be recommended. The phenotype should therefore be regarded as a hypothesis-generating framework for future research rather than as an established clinical entity. Observed combinations of higher FGF23, lower soluble Klotho, CKD, LVH, and HFpEF features may reflect shared dependence on kidney dysfunction, age, inflammation, phosphate burden, anemia, or treatment exposure rather than a distinct causal endotype. Accordingly, neither should currently be regarded as a standardized biomarker for routine clinical use or as an established tool for diagnosis, prognosis, treatment selection, or response monitoring. Likewise, direct FGF23-, FGFR4-, or Klotho-directed interventions remain investigational and should not be considered validated therapeutic options. The investigational framework presented in **Figure 5B** is intended for hypothesis generation, prospective biomarker and phenotype validation, mechanistic phenotyping, and trial design rather than for routine clinical decision-making. Clinical management should continue to follow established assessments and guideline-supported therapies for HF, CKD, diabetes, obesity, and CKD-mineral and bone disorder.

## Conclusion

CKM-related heart failure is a multisystem endocrine-metabolic disorder in which kidney, metabolic, vascular, and cardiac abnormalities interact before overt decompensation. Within this context, the FGF23-Klotho axis provides a biologically plausible, hypothesis-generating research framework linking kidney and mineral stress with adverse cardiac phenotypes. However, human evidence remains predominantly observational, and direct cardiac mechanisms are supported mainly by preclinical studies. Obesity, insulin resistance, RAAS activation, anemia, gut-derived metabolites, and cardiometabolic therapies should therefore be interpreted as modifiers, competing pathways, confounders, or treatment contexts rather than as evidence that the FGF23-Klotho axis mediates CKM-related heart failure or therapeutic benefit.

Circulating FGF23 and soluble Klotho are not standardized biomarkers for routine clinical assessment, their incremental value beyond established kidney, mineral, cardiac, and imaging measures remains unproven, and the proposed mineral-endocrine-enriched phenotype lacks diagnostic criteria, validated biomarker thresholds, and prospective validation. Direct FGF23-, FGFR4-, or Klotho-directed therapies also remain investigational. Accordingly, the FGF23-Klotho axis should currently be regarded as a research framework with potential mechanistic and translational value rather than as a mature clinical assessment or treatment-selection tool. Current care should continue to follow guideline-supported management of HF, CKD, diabetes, obesity, and CKD-mineral and bone disorder, while future research should prioritize assay harmonization, prospective phenotype validation, formal evaluation of incremental value, and carefully selected pathway-directed trials.
