# Supplementary material for: Endocrine crosstalk in cardiovascular-kidney-metabolic syndrome-related heart failure: the fibroblast growth factor 23-Klotho axis and cardiometabolic therapy
Source: Front Endocrinol (Lausanne). 2026 Sep 8;17:1933732. doi: 10.3389/fendo.2026.1933732 (PMC13594805; doi:10.3389/fendo.2026.1933732)
Supplement: Supplementary file 1 [file Table1.docx]

**Supplementary Table S1. Complete database-specific search strategies, search dates, and record counts**

| **Database** | **Search block** | **Complete search string** | **Search date** | **Date range** | **Limits / screening rule** | **Records identified or screened** | **Purpose in review** |
| --- | --- | --- | --- | --- | --- | --- | --- |
| PubMed/MEDLINE | Q1. CKM syndrome and heart failure | (("cardiovascular-kidney-metabolic syndrome"[Title/Abstract] OR "CKM syndrome"[Title/Abstract] OR "cardiovascular-kidney-metabolic health"[Title/Abstract]) AND ("heart failure"[Title/Abstract] OR HFpEF[Title/Abstract] OR HFrEF[Title/Abstract])) AND (1000/01/01:2026/07/18[Date - Publication]) | 18 July 2026 | Database inception to 18 July 2026 | Title/Abstract fields; publication date capped at 18 July 2026; no article-type restriction at retrieval. | 140 | CKM framework, CKM staging and heart failure framing. |
| PubMed/MEDLINE | Q2. Heart failure phenotypes in cardiometabolic-kidney disease | (("heart failure with preserved ejection fraction"[Title/Abstract] OR HFpEF[Title/Abstract] OR "heart failure with reduced ejection fraction"[Title/Abstract] OR HFrEF[Title/Abstract]) AND (obesity[Title/Abstract] OR diabetes[Title/Abstract] OR "chronic kidney disease"[Title/Abstract] OR CKD[Title/Abstract] OR cardiorenal[Title/Abstract])) AND (1000/01/01:2026/07/18[Date - Publication]) | 18 July 2026 | Database inception to 18 July 2026 | Title/Abstract fields; publication date capped at 18 July 2026; no article-type restriction at retrieval. | 3416 | HFpEF/HFrEF phenotype framing in diabetes, obesity, CKD and cardiorenal disease. |
| PubMed/MEDLINE | Q3. FGF23-Klotho, CKD/DKD and cardiac remodeling | ((FGF23[Title/Abstract] OR "fibroblast growth factor 23"[Title/Abstract]) AND (Klotho[Title/Abstract] OR "soluble Klotho"[Title/Abstract]) AND ("chronic kidney disease"[Title/Abstract] OR CKD[Title/Abstract] OR "diabetic kidney disease"[Title/Abstract] OR DKD[Title/Abstract]) AND ("heart failure"[Title/Abstract] OR "left ventricular hypertrophy"[Title/Abstract] OR LVH[Title/Abstract] OR "cardiac fibrosis"[Title/Abstract])) AND (1000/01/01:2026/07/18[Date - Publication]) | 18 July 2026 | Database inception to 18 July 2026 | Title/Abstract fields; publication date capped at 18 July 2026; no article-type restriction at retrieval. | 74 | FGF23-Klotho endocrine bridge, kidney-mineral metabolism and cardiac remodeling. |
| PubMed/MEDLINE | Q4. Metabolic and adipose drivers of HFpEF | (("insulin resistance"[Title/Abstract] OR hyperglycemia[Title/Abstract] OR obesity[Title/Abstract] OR "adipose tissue dysfunction"[Title/Abstract] OR adipokines[Title/Abstract]) AND (HFpEF[Title/Abstract] OR "heart failure with preserved ejection fraction"[Title/Abstract])) AND (1000/01/01:2026/07/18[Date - Publication]) | 18 July 2026 | Database inception to 18 July 2026 | Title/Abstract fields; publication date capped at 18 July 2026; no article-type restriction at retrieval. | 1275 | Insulin resistance, hyperglycemia, obesity-related HFpEF and adipose inflammation. |
| PubMed/MEDLINE | Q5. Cardiometabolic therapies | (("SGLT2 inhibitors"[Title/Abstract] OR "sodium-glucose cotransporter 2"[Title/Abstract] OR "GLP-1 receptor agonists"[Title/Abstract] OR "glucagon-like peptide-1"[Title/Abstract] OR finerenone[Title/Abstract] OR "mineralocorticoid receptor antagonist"[Title/Abstract] OR "weight loss"[Title/Abstract]) AND ("heart failure"[Title/Abstract] OR HFpEF[Title/Abstract] OR CKD[Title/Abstract] OR "diabetic kidney disease"[Title/Abstract])) AND (1000/01/01:2026/07/18[Date - Publication]) | 18 July 2026 | Database inception to 18 July 2026 | Title/Abstract fields; publication date capped at 18 July 2026; no article-type restriction at retrieval. | 10059 | SGLT2 inhibitors, GLP-1 receptor agonists, finerenone, MRAs and weight-loss therapies. |
| PubMed/MEDLINE | Q6. Gut-derived endocrine-metabolic signals | (("gut microbiota"[Title/Abstract] OR microbiome[Title/Abstract] OR TMAO[Title/Abstract] OR "trimethylamine N-oxide"[Title/Abstract] OR "short-chain fatty acids"[Title/Abstract] OR "bile acids"[Title/Abstract]) AND ("heart failure"[Title/Abstract] OR "cardiovascular disease"[Title/Abstract] OR CKD[Title/Abstract] OR "chronic kidney disease"[Title/Abstract])) AND (1000/01/01:2026/07/18[Date - Publication]) | 18 July 2026 | Database inception to 18 July 2026 | Title/Abstract fields; publication date capped at 18 July 2026; no article-type restriction at retrieval. | 4561 | Gut microbiota-derived metabolites and CKM-related endocrine-metabolic signaling. |
| Web of Science Core Collection | WoS-Q1. CKM syndrome and heart failure | TS=(("cardiovascular-kidney-metabolic syndrome" OR "CKM syndrome" OR "cardiovascular-kidney-metabolic health") AND ("heart failure" OR HFpEF OR HFrEF)) | 18 July 2026 | Database inception to 18 July 2026 | Advanced Search; TS= topic query; Timespan set to All years/database inception; no document-type restriction at retrieval. | 173 | CKM framework, CKM staging and heart failure framing. |
| Web of Science Core Collection | WoS-Q2. Heart failure phenotypes in cardiometabolic-kidney disease | TS=(("heart failure with preserved ejection fraction" OR HFpEF OR "heart failure with reduced ejection fraction" OR HFrEF) AND (obesity OR diabetes OR "chronic kidney disease" OR CKD OR cardiorenal)) | 18 July 2026 | Database inception to 18 July 2026 | Advanced Search; TS= topic query; Timespan set to All years/database inception; no document-type restriction at retrieval. | 3902 | HFpEF/HFrEF phenotype framing in diabetes, obesity, CKD and cardiorenal disease. |
| Web of Science Core Collection | WoS-Q3. FGF23-Klotho, CKD/DKD and cardiac remodeling | TS=((FGF23 OR "fibroblast growth factor 23") AND (Klotho OR "soluble Klotho") AND ("chronic kidney disease" OR CKD OR "diabetic kidney disease" OR DKD) AND ("heart failure" OR "left ventricular hypertrophy" OR LVH OR "cardiac fibrosis")) | 18 July 2026 | Database inception to 18 July 2026 | Advanced Search; TS= topic query; Timespan set to All years/database inception; no document-type restriction at retrieval. | 196 | FGF23-Klotho endocrine bridge, kidney-mineral metabolism and cardiac remodeling. |
| Web of Science Core Collection | WoS-Q4. Metabolic and adipose drivers of HFpEF | TS=(("insulin resistance" OR hyperglycemia OR obesity OR "adipose tissue dysfunction" OR adipokines) AND (HFpEF OR "heart failure with preserved ejection fraction")) | 18 July 2026 | Database inception to 18 July 2026 | Advanced Search; TS= topic query; Timespan set to All years/database inception; no document-type restriction at retrieval. | 1547 | Insulin resistance, hyperglycemia, obesity-related HFpEF and adipose inflammation. |
| Web of Science Core Collection | WoS-Q5. Cardiometabolic therapies | TS=(("SGLT2 inhibitors" OR "sodium-glucose cotransporter 2" OR "GLP-1 receptor agonists" OR "glucagon-like peptide-1" OR finerenone OR "mineralocorticoid receptor antagonist" OR "weight loss") AND ("heart failure" OR HFpEF OR CKD OR "diabetic kidney disease")) | 18 July 2026 | Database inception to 18 July 2026 | Advanced Search; TS= topic query; Timespan set to All years/database inception; no document-type restriction at retrieval. | 12775 | SGLT2 inhibitors, GLP-1 receptor agonists, finerenone, MRAs and weight-loss therapies. |
| Web of Science Core Collection | WoS-Q6. Gut-derived endocrine-metabolic signals | TS=(("gut microbiota" OR microbiome OR TMAO OR "trimethylamine N-oxide" OR "short-chain fatty acids" OR "bile acids") AND ("heart failure" OR "cardiovascular disease" OR CKD OR "chronic kidney disease")) | 18 July 2026 | Database inception to 18 July 2026 | Advanced Search; TS= topic query; Timespan set to All years/database inception; no document-type restriction at retrieval. | 7097 | Gut microbiota-derived metabolites and CKM-related endocrine-metabolic signaling. |
| Google Scholar / citation tracking | GS-Q1. CKM syndrome and heart failure | "cardiovascular-kidney-metabolic syndrome" "heart failure" OR "CKM syndrome" "heart failure" OR "cardiovascular-kidney-metabolic health" "heart failure" | 18 July 2026 | Database inception to 18 July 2026 | No custom year restriction; first 200 relevance-ranked results screened per concept block; citation tracking and reference-list checking used as supplementary identification. | 200 | CKM framework, CKM staging and heart failure framing. |
| Google Scholar / citation tracking | GS-Q2. Heart failure phenotypes in cardiometabolic-kidney disease | "heart failure with preserved ejection fraction" OR HFpEF OR "heart failure with reduced ejection fraction" OR HFrEF obesity diabetes "chronic kidney disease" cardiorenal | 18 July 2026 | Database inception to 18 July 2026 | No custom year restriction; first 200 relevance-ranked results screened per concept block; citation tracking and reference-list checking used as supplementary identification. | 200 | HFpEF/HFrEF phenotype framing in diabetes, obesity, CKD and cardiorenal disease. |
| Google Scholar / citation tracking | GS-Q3. FGF23-Klotho, CKD/DKD and cardiac remodeling | FGF23 Klotho "chronic kidney disease" "diabetic kidney disease" "heart failure" "left ventricular hypertrophy" "cardiac fibrosis" | 18 July 2026 | Database inception to 18 July 2026 | No custom year restriction; first 200 relevance-ranked results screened per concept block; citation tracking and reference-list checking used as supplementary identification. | 200 | FGF23-Klotho endocrine bridge, kidney-mineral metabolism and cardiac remodeling. |
| Google Scholar / citation tracking | GS-Q4. Metabolic and adipose drivers of HFpEF | "insulin resistance" hyperglycemia obesity "adipose tissue dysfunction" adipokines HFpEF "heart failure with preserved ejection fraction" | 18 July 2026 | Database inception to 18 July 2026 | No custom year restriction; first 200 relevance-ranked results screened per concept block; citation tracking and reference-list checking used as supplementary identification. | 200 | Insulin resistance, hyperglycemia, obesity-related HFpEF and adipose inflammation. |
| Google Scholar / citation tracking | GS-Q5. Cardiometabolic therapies | "SGLT2 inhibitors" "GLP-1 receptor agonists" finerenone "mineralocorticoid receptor antagonist" "weight loss" "heart failure" HFpEF CKD "diabetic kidney disease" | 18 July 2026 | Database inception to 18 July 2026 | No custom year restriction; first 200 relevance-ranked results screened per concept block; citation tracking and reference-list checking used as supplementary identification. | 200 | SGLT2 inhibitors, GLP-1 receptor agonists, finerenone, MRAs and weight-loss therapies. |
| Google Scholar / citation tracking | GS-Q6. Gut-derived endocrine-metabolic signals | "gut microbiota" microbiome TMAO "trimethylamine N-oxide" "short-chain fatty acids" "bile acids" "heart failure" "cardiovascular disease" CKD "chronic kidney disease" | 18 July 2026 | Database inception to 18 July 2026 | No custom year restriction; first 200 relevance-ranked results screened per concept block; citation tracking and reference-list checking used as supplementary identification. | 200 | Gut microbiota-derived metabolites and CKM-related endocrine-metabolic signaling. |
| PubMed/MEDLINE | Q7. Phosphate/CKD-MBD interventions and cardiovascular outcomes (targeted supplementary topic search) | (("Hyperphosphatemia"[MeSH Terms] OR hyperphosphatemia[Title/Abstract] OR phosphate[Title/Abstract] OR "phosphate binder"[Title/Abstract] OR "phosphate binders"[Title/Abstract] OR cinacalcet[Title/Abstract] OR "lanthanum carbonate"[Title/Abstract] OR sevelamer[Title/Abstract] OR "CKD-MBD"[Title/Abstract] OR "chronic kidney disease-mineral and bone disorder"[Title/Abstract]) AND ("Cardiovascular Diseases"[MeSH Terms] OR cardiovascular[Title/Abstract] OR "cardiovascular outcomes"[Title/Abstract] OR "heart failure"[Title/Abstract] OR mortality[Title/Abstract] OR "vascular calcification"[Title/Abstract]) AND ("Kidney Diseases, Chronic"[MeSH Terms] OR "chronic kidney disease"[Title/Abstract] OR CKD[Title/Abstract] OR dialysis[Title/Abstract] OR hemodialysis[Title/Abstract])) AND (1000/01/01:2026/07/18[Date - Publication]) | 25 July 2026 | Database inception to 18 July 2026 | MeSH plus Title/Abstract fields; publication date capped at 18 July 2026; no article-type restriction. Broad topic search used for literature identification; DOI/exact-title verification was used only to confirm the four retained reports. DOI/exact-title verification confirmed refs. 101-104 in PubMed: PMID 23121374, 22822075, 32917784, and 34003226. | 4736 | Phosphate lowering, cinacalcet, phosphate binders, vascular end points, and cardiovascular outcomes in CKD/dialysis; refs. 101-104. |
| Web of Science Core Collection | WoS-Q7. Phosphate/CKD-MBD interventions and cardiovascular outcomes (topic cross-check) | TS=((hyperphosphatemia OR phosphate OR "phosphate binder*" OR cinacalcet OR "lanthanum carbonate" OR sevelamer OR "CKD-MBD" OR "chronic kidney disease-mineral and bone disorder") AND (cardiovascular OR "cardiovascular outcomes" OR "heart failure" OR mortality OR "vascular calcification") AND ("chronic kidney disease" OR CKD OR dialysis OR hemodialysis)) | 25 July 2026 | Database inception to 18 July 2026 | Advanced Search using TS= topic fields; timespan set to all years/database inception. Used as cross-database topic verification and citation-context checking; not pooled separately to avoid double-counting records already identified through the PubMed/MEDLINE Q7 topic search. | Cross-check only; not pooled separately | Cross-database verification of phosphate/CKD-MBD cardiovascular-outcomes literature, including EVOLVE, Block et al., IMPROVE-CKD, and LANDMARK. |
| Google Scholar / citation tracking | GS-Q7. Phosphate/CKD-MBD interventions and cardiovascular outcomes (topic/citation check) | ("hyperphosphatemia" OR phosphate OR "phosphate binder" OR cinacalcet OR "lanthanum carbonate" OR sevelamer OR "CKD-MBD") ("cardiovascular outcomes" OR "heart failure" OR mortality OR "vascular calcification") ("chronic kidney disease" OR CKD OR dialysis OR hemodialysis) | 25 July 2026 | Database inception to 18 July 2026 | Broad topic search plus backward/forward citation tracking. Used to verify relevance and citation context for the four retained reports; not pooled separately to avoid double-counting. | Citation cross-check only; not pooled separately | Supplementary citation-context verification for refs. 101-104. |
| PubMed/MEDLINE | Q8. Sex differences / heterogeneity in FGF23, CKD progression, and HF treatment response (targeted supplementary topic search) | (((FGF23[Title/Abstract] OR "fibroblast growth factor 23"[Title/Abstract]) AND ("coronary heart disease"[Title/Abstract] OR "Coronary Heart Disease"[MeSH Terms] OR CHD[Title/Abstract]) AND (sex[Title/Abstract] OR gender[Title/Abstract] OR heterogeneity[Title/Abstract] OR community-living[Title/Abstract])) OR (("CKD progression"[Title/Abstract] OR "chronic kidney disease"[Title/Abstract] OR CKD[Title/Abstract]) AND mortality[Title/Abstract] AND (men[Title/Abstract] OR women[Title/Abstract] OR sex[Title/Abstract]) AND (Sweden[Title/Abstract] OR nationwide[Title/Abstract])) OR ((dapagliflozin[Title/Abstract] OR DAPA-HF[Title/Abstract] OR DELIVER[Title/Abstract]) AND "heart failure"[Title/Abstract] AND (sex[Title/Abstract] OR women[Title/Abstract] OR men[Title/Abstract] OR "ejection fraction"[Title/Abstract] OR "treatment response"[Title/Abstract]))) AND (1000/01/01:2026/07/18[Date - Publication]) | 25 July 2026 | Database inception to 18 July 2026 | Title/Abstract plus selected MeSH phrase for coronary heart disease; no article-type restriction. PMID/DOI/exact-title checks used only to verify retained reports. Covered PMIDs: 29516098, 33434591, 36342789. | 1155 | Sex-specific effect modification and outcome heterogeneity relevant to FGF23-associated coronary disease, CKD progression/mortality, and dapagliflozin response across EF phenotypes; refs. 105-107. |
| Web of Science Core Collection | WoS-Q8. Sex differences / heterogeneity in FGF23, CKD progression, and HF treatment response (topic cross-check) | TS=(((FGF23 OR "fibroblast growth factor 23") AND ("coronary heart disease" OR CHD) AND (sex OR gender OR heterogeneity OR "community-living")) OR (("CKD progression" OR "chronic kidney disease" OR CKD) AND mortality AND (men OR women OR sex) AND (Sweden OR nationwide)) OR ((dapagliflozin OR "DAPA-HF" OR DELIVER) AND "heart failure" AND (sex OR women OR men OR "ejection fraction" OR "treatment response"))) | 25 July 2026 | Database inception to 18 July 2026 | Topic-field cross-check only; not pooled separately to avoid double counting with PubMed Q8. | Cross-check only; not pooled separately | Cross-database verification of sex/heterogeneity literature context, including Panwar et al., Swartling et al., and Wang et al. |
| Google Scholar / citation tracking | GS-Q8. Sex differences / heterogeneity in FGF23, CKD progression, and HF treatment response (topic/citation check) | ((FGF23 OR "fibroblast growth factor 23") "coronary heart disease" (sex OR gender OR heterogeneity)) OR ("CKD progression" mortality (men OR women) Sweden nationwide) OR (dapagliflozin "heart failure" sex "ejection fraction" DAPA-HF DELIVER) | 25 July 2026 | Database inception to 18 July 2026 | Supplementary citation-context check; screened only to verify citation context and forward/backward linkage for newly added refs. | Citation cross-check only; not pooled separately | Supplementary citation-context verification for refs. 105-107. |
| Total | Initial six concept blocks plus Q7 phosphate/CKD-MBD and Q8 sex/heterogeneity supplementary topic searches | Six original concept blocks across PubMed/MEDLINE, Web of Science Core Collection, and Google Scholar/citation tracking, plus PubMed/MEDLINE Q7 topic search for phosphate/CKD-MBD interventions and cardiovascular outcomes and PubMed/MEDLINE Q8 topic search for sex differences/heterogeneity in FGF23, CKD progression, and heart-failure treatment response. | 25 July 2026 | Database inception to 18 July 2026 | All database searches were reconstructed for the revision-stage audit. Q7 and Q8 targeted supplementary topic searches were executed on 25 July 2026 with date coverage from database inception to 18 July 2026; DOI/PMID/exact-title checks were used only for verification. | 52306 | Final references included in the structured narrative review: n = 107; duplicate records removed: n = 18,642; title/abstract screened: n = 33,664; full-text assessed: n = 319; full-text excluded: n = 212. Newly retained reports: refs. 101-107. |
