# Supplementary material for: Endocrine crosstalk in cardiovascular-kidney-metabolic syndrome-related heart failure: the fibroblast growth factor 23-Klotho axis and cardiometabolic therapy
Source: Front Endocrinol (Lausanne). 2026 Sep 8;17:1933732. doi: 10.3389/fendo.2026.1933732 (PMC13594805; doi:10.3389/fendo.2026.1933732)
Supplement: Supplementary file 2 [file Table2.docx]

**Supplementary Table S2.** Study-level methodological appraisal and assignment to evidence tiers.

| **Ref.** | **Citation** | **Study design** | **Role in review** | **Appraisal tool** | **Overall appraisal** | **Key limitations / basis for judgment** | **Evidence tier** |
| --- | --- | --- | --- | --- | --- | --- | --- |
| 1 | Ndumele CE et al., 2023: Cardiovascular- Kidney-Metabolic Health: A Presidential Advisory From the American Heart Association | Scientific advisory/consensus framework | Background/framework | Contextual source; not included in study-level RoB grading | Authoritative contextual source | Defines CKM/HF concepts or risk framework; does not independently establish an intervention effect. | Contextual |
| 2 | Heidenreich PA et al., 2022: 2022 AHA/ACC/HFSA Guideline for the Management of Heart Failure: A Report of the American College of Cardiology/American Heart Association Joint Committee on Clinical Practice Guidelines | Clinical practice guideline/focused guideline update | Guideline/measurement standard | AGREE II | High methodological confidence | Recommendations are evidence-based, but applicability depends on population, date, and scope. | Tier 1 / established clinical context |
| 3 | Bozkurt B et al., 2021: Universal definition and classification of heart failure: a report of the Heart Failure Society of America, Heart Failure Association of the European Society of Cardiology, Japanese Heart Failure Society and Writing Committee of the Universal Definition of Heart Failure | Scientific advisory/consensus framework | Background/framework | Contextual source; not included in study-level RoB grading | Authoritative contextual source | Defines CKM/HF concepts or risk framework; does not independently establish an intervention effect. | Contextual |
| 4 | McDonagh TA et al., 2021: 2021 ESC Guidelines for the diagnosis and treatment of acute and chronic heart failure | Clinical practice guideline/focused guideline update | Guideline/measurement standard | AGREE II | High methodological confidence | Recommendations are evidence-based, but applicability depends on population, date, and scope. | Tier 1 / established clinical context |
| 5 | McDonagh TA et al., 2023: 2023 Focused Update of the 2021 ESC Guidelines for the diagnosis and treatment of acute and chronic heart failure | Clinical practice guideline/focused guideline update | Guideline/measurement standard | AGREE II | High methodological confidence | Recommendations are evidence-based, but applicability depends on population, date, and scope. | Tier 1 / established clinical context |
| 6 | Redfield MM et al., 2023: Heart Failure With Preserved Ejection Fraction: A Review | Narrative/state-of-the-art review | Background/framework | SANRA (contextual appraisal only) | Contextual synthesis; not used alone to determine certainty | Secondary source; conclusions depend on the underlying primary evidence. | Contextual |
| 7 | Pieske B et al., 2020: How to Diagnose Heart Failure with Preserved Ejection Fraction: The HFA-PEFF Diagnostic Algorithm: A Consensus Recommendation from the Heart Failure Association of the European Society of Cardiology | Expert consensus/recommendation statement | Guideline/measurement standard | AGREE II-GRS | Moderate-to-high methodological confidence | Consensus-based elements and indirect evidence remain. | Tier 1 for diagnostic/measurement standards |
| 8 | Reddy YNV et al., 2018: A simple, evidence-based approach to help guide diagnosis of heart failure with preserved ejection fraction | Diagnostic prediction model development/validation | Human association, risk stratification, or phenotyping | PROBAST | Some concerns | Referral-population derivation and spectrum/applicability concerns; requires external validation across settings. | Tier 2 / translational |
| 9 | Paulus WJ et al., 2013: A novel paradigm for heart failure with preserved ejection fraction: comorbidities drive myocardial dysfunction and remodeling through coronary microvascular endothelial inflammation | Narrative/state-of-the-art review | Background/framework | SANRA (contextual appraisal only) | Contextual synthesis; not used alone to determine certainty | Secondary source; conclusions depend on the underlying primary evidence. | Contextual |
| 10 | Owan TE et al., 2006: Trends in prevalence and outcome of heart failure with preserved ejection fraction | Observational cohort study | Human association, risk stratification, or phenotyping | QUIPS | Moderate risk of bias | Residual confounding, selection, exposure measurement, and model-dependence limit causal inference. | Tier 2 / translational |
| 11 | Bhatia RS et al., 2006: Outcome of heart failure with preserved ejection fraction in a population-based study | Observational cohort study | Human association, risk stratification, or phenotyping | QUIPS | Moderate risk of bias | Residual confounding, selection, exposure measurement, and model-dependence limit causal inference. | Tier 2 / translational |
| 12 | Borlaug BA et al., 2010: Global cardiovascular reserve dysfunction in heart failure with preserved ejection fraction | Physiological or imaging case-control/cross-sectional study | Human association, risk stratification, or phenotyping | JBI analytical cross-sectional | Moderate risk of bias | Selected referral samples, modest sample size, and cross-sectional design limit generalizability and causality. | Tier 2 / translational |
| 13 | Mohammed SF et al., 2015: Coronary Microvascular Rarefaction and Myocardial Fibrosis in Heart Failure With Preserved Ejection Fraction | Autopsy case-control study | Human association, risk stratification, or phenotyping | JBI case-control | Moderate-to-high risk of bias | Post-mortem selection, retrospective phenotype ascertainment, and confounding limit causal inference. | Tier 2 / translational |
| 14 | Nagueh SF et al., 2016: Recommendations for the Evaluation of Left Ventricular Diastolic Function by Echocardiography: An Update from the American Society of Echocardiography and the European Association of Cardiovascular Imaging | Expert consensus/recommendation statement | Guideline/measurement standard | AGREE II-GRS | Moderate-to-high methodological confidence | Consensus-based elements and indirect evidence remain. | Tier 1 for diagnostic/measurement standards |
| 15 | Edmonston D et al., 2024: FGF23 and klotho at the intersection of kidney and cardiovascular disease | Narrative/state-of-the-art review | Background/framework | SANRA (contextual appraisal only) | Contextual synthesis; not used alone to determine certainty | Secondary source; conclusions depend on the underlying primary evidence. | Contextual |
| 16 | Ho BB et al., 2021: FGF23 signalling and physiology | Narrative/state-of-the-art review | Background/framework | SANRA (contextual appraisal only) | Contextual synthesis; not used alone to determine certainty | Secondary source; conclusions depend on the underlying primary evidence. | Contextual |
| 17 | Faul C et al., 2011: FGF23 induces left ventricular hypertrophy | Preclinical animal/in vitro mechanistic study | Mechanistic plausibility / hypothesis generation | SYRCLE RoB (animal components) + predefined in-vitro reporting criteria | Some concerns / unclear risk | Seminal animal and cellular evidence supports biological plausibility, but allocation concealment, blinding, and sample-size justification were incompletely reported; directness to human CKM-related HF is very limited. | Tier 3 / mechanistic or hypothesis-generating |
| 18 | Gutiérrez OM et al., 2009: Fibroblast growth factor 23 and left ventricular hypertrophy in chronic kidney disease | Cross-sectional/observational CKD study | Human association, risk stratification, or phenotyping | JBI analytical cross-sectional | Moderate risk of bias | Cross-sectional association; residual confounding by kidney function, phosphate metabolism, inflammation, and treatment exposure, together with reverse causation, limits causal interpretation. | Tier 2 / translational |
| 19 | Nakano T et al., 2023: Direct and indirect effects of fibroblast growth factor 23 on the heart | Narrative/state-of-the-art review | Background/framework | SANRA (contextual appraisal only) | Contextual synthesis; not used alone to determine certainty | Secondary source; conclusions depend on the underlying primary evidence. | Contextual |
| 20 | Anker SD et al., 2021: Empagliflozin in Heart Failure with a Preserved Ejection Fraction | Randomized controlled trial | Clinical efficacy/safety | Cochrane RoB 2 | Low risk of bias | The primary benefit was driven mainly by fewer heart-failure hospitalizations; mortality benefit and direct effects on FGF23-Klotho biology were not established. | Tier 1 / established clinical evidence |
| 21 | Bakris GL et al., 2020: Effect of Finerenone on Chronic Kidney Disease Outcomes in Type 2 Diabetes | Randomized controlled trial | Clinical efficacy/safety | Cochrane RoB 2 | Low risk of bias | Findings apply to albuminuric type 2 diabetes with CKD; heart-failure outcomes were not the primary focus, and potassium monitoring is required. | Tier 1 / established clinical evidence |
| 22 | Heerspink HJL et al., 2020: Dapagliflozin in Patients with Chronic Kidney Disease | Randomized controlled trial | Clinical efficacy/safety | Cochrane RoB 2 | Low risk of bias | The trial established kidney and cardiovascular benefit in CKD, but direct extrapolation to HF phenotypes or FGF23-Klotho modulation remains indirect. | Tier 1 / established clinical evidence |
| 23 | Kosiborod MN et al., 2023: Semaglutide in Patients with Heart Failure with Preserved Ejection Fraction and Obesity | Randomized controlled trial | Clinical efficacy/safety | Cochrane RoB 2 | Low risk of bias | The enrolled population had obesity-related HFpEF; benefits concerned symptoms, function, and weight and should not be extrapolated to lean or non-obesity-related HFpEF. | Tier 1 / established clinical evidence |
| 24 | Pitt B et al., 2021: Cardiovascular Events with Finerenone in Kidney Disease and Type 2 Diabetes | Randomized controlled trial | Clinical efficacy/safety | Cochrane RoB 2 | Low risk of bias | Findings apply to type 2 diabetes with CKD and were driven by cardiovascular events including heart-failure hospitalization; direct HFpEF-specific inference is limited. | Tier 1 / established clinical evidence |
| 25 | Solomon SD et al., 2022: Dapagliflozin in Heart Failure with Mildly Reduced or Preserved Ejection Fraction | Randomized controlled trial | Clinical efficacy/safety | Cochrane RoB 2 | Low risk of bias | The principal benefit was reduced worsening heart failure; effects on mortality and endocrine mechanisms were not established. | Tier 1 / established clinical evidence |
| 26 | Khan SS et al., 2023: Novel Prediction Equations for Absolute Risk Assessment of Total Cardiovascular Disease Incorporating Cardiovascular-Kidney-Metabolic Health: A Scientific Statement From the American Heart Association | Scientific advisory/consensus framework | Background/framework | Contextual source; not included in study-level RoB grading | Authoritative contextual source | Defines CKM/HF concepts or risk framework; does not independently establish an intervention effect. | Contextual |
| 27 | Khan SS et al., 2024: Development and Validation of the American Heart Association's PREVENT Equations | Prognostic prediction model development and external validation | Human association, risk stratification, or phenotyping | PROBAST | Low risk to some concerns | Large multi-cohort validation, but transportability and calibration require local evaluation. | Tier 2 / translational |
| 28 | Kidney Disease: Improving Global Outcomes CKD Work Group,2024: KDIGO 2024 Clinical Practice Guideline for the Evaluation and Management of Chronic Kidney Disease | Clinical practice guideline/focused guideline update | Guideline/measurement standard | AGREE II | High methodological confidence | Recommendations are evidence-based, but applicability depends on population, date, and scope. | Tier 1 / established clinical context |
| 29 | Go AS et al., 2004: Chronic kidney disease and the risks of death, cardiovascular events, and hospitalization | Observational cohort study | Human association, risk stratification, or phenotyping | QUIPS | Moderate risk of bias | Residual confounding, selection, exposure measurement, and model-dependence limit causal inference. | Tier 2 / translational |
| 30 | Chronic Kidney Disease Prognosis Consortium,2010: Association of estimated glomerular filtration rate and albuminuria with all-cause and cardiovascular mortality in general population cohorts: a collaborative meta-analysis | Collaborative meta-analysis of observational cohorts | Human association, risk stratification, or phenotyping | JBI Critical Appraisal Checklist for Systematic Reviews and Research Syntheses | Moderate confidence | Observational evidence remains vulnerable to residual confounding despite large size and consistency. | Tier 2 / established epidemiologic association |
| 31 | Gansevoort RT et al., 2013: Chronic kidney disease and cardiovascular risk: epidemiology, mechanisms, and prevention | Narrative/state-of-the-art review | Background/framework | SANRA (contextual appraisal only) | Contextual synthesis; not used alone to determine certainty | Secondary source; conclusions depend on the underlying primary evidence. | Contextual |
| 32 | Claudel SE et al., 2025: Albuminuria in Cardiovascular, Kidney, and Metabolic Disorders: A State- of-the-Art Review | Narrative/state-of-the-art review | Background/framework | SANRA (contextual appraisal only) | Contextual synthesis; not used alone to determine certainty | Secondary source; conclusions depend on the underlying primary evidence. | Contextual |
| 33 | Almahmoud MF et al., 2018: Fibroblast Growth Factor-23 and Heart Failure With Reduced Versus Preserved Ejection Fraction: MESA | Observational cohort study | Human association, risk stratification, or phenotyping | QUIPS | Moderate risk of bias | Single baseline FGF23 measurement and relatively few adjudicated HF-subtype events limit precision; residual confounding and assay-related variability remain. | Tier 2 / translational |
| 34 | Binnenmars SH et al., 2022: Fibroblast Growth Factor 23 and Risk of New Onset Heart Failure With Preserved or Reduced Ejection Fraction: The PREVEND Study | Observational cohort study | Human association, risk stratification, or phenotyping | QUIPS | Moderate risk of bias | Single baseline biomarker measurement, population selection, and residual confounding limit causal inference and generalizability across CKD severity. | Tier 2 / translational |
| 35 | Leidner AS et al., 2023: Fibroblast Growth Factor 23 and Risk of Heart Failure Subtype: The CRIC (Chronic Renal Insufficiency Cohort) Study | Observational cohort study | Human association, risk stratification, or phenotyping | QUIPS | Moderate risk of bias | The CKD-enriched cohort improves relevance to renal disease but limits generalizability; residual confounding by CKD severity, mineral metabolism, and treatment remains. | Tier 2 / translational |
| 36 | Perkovic V et al., 2024: Effects of Semaglutide on Chronic Kidney Disease in Patients with Type 2 Diabetes | Randomized controlled trial | Clinical efficacy/safety | Cochrane RoB 2 | Low risk of bias | The trial applies to type 2 diabetes with CKD; kidney outcomes were primary, while direct HF-specific and FGF23-Klotho effects remain secondary or untested. | Tier 1 / established clinical evidence |
| 37 | Solomon SD et al., 2024: Finerenone in Heart Failure with Mildly Reduced or Preserved Ejection Fraction | Randomized controlled trial | Clinical efficacy/safety | Cochrane RoB 2 | Low risk of bias | Evidence is currently dominated by one pivotal HFmrEF/HFpEF trial; replication and longer-term safety data across broader phenotypes remain limited. | Tier 1 / established clinical evidence |
| 38 | The EMPA-KIDNEY Collaborative Group et al., 2023: Empagliflozin in Patients with Chronic Kidney Disease | Randomized controlled trial | Clinical efficacy/safety | Cochrane RoB 2 | Low risk of bias | The broad CKD population and early trial termination support kidney benefit but limit detailed HF-phenotype and mechanistic inference. | Tier 1 / established clinical evidence |
| 39 | Ketteler M et al., 2017: Executive summary of the 2017 KDIGO Chronic Kidney Disease-Mineral and Bone Disorder Guideline Update: what's changed and why it matters | Clinical practice guideline/focused guideline update | Guideline/measurement standard | AGREE II | High methodological confidence | Recommendations are evidence-based, but applicability depends on population, date, and scope. | Tier 1 / established clinical context |
| 40 | Kidney Disease: Improving Global Outcomes Diabetes Work Group,2022: KDIGO 2022 Clinical Practice Guideline for Diabetes Management in Chronic Kidney Disease | Clinical practice guideline/focused guideline update | Guideline/measurement standard | AGREE II | High methodological confidence | Recommendations are evidence-based, but applicability depends on population, date, and scope. | Tier 1 / established clinical context |
| 41 | Jia G et al., 2018: Diabetic Cardiomyopathy: An Update of Mechanisms Contributing to This Clinical Entity | Narrative/state-of-the-art review | Background/framework | SANRA (contextual appraisal only) | Contextual synthesis; not used alone to determine certainty | Secondary source; conclusions depend on the underlying primary evidence. | Contextual |
| 42 | Ritchie RH et al., 2020: Basic mechanisms of diabetic heart disease | Narrative/state-of-the-art review | Background/framework | SANRA (contextual appraisal only) | Contextual synthesis; not used alone to determine certainty | Secondary source; conclusions depend on the underlying primary evidence. | Contextual |
| 43 | Levelt E et al., 2016: Relationship Between Left Ventricular Structural and Metabolic Remodeling in Type 2 Diabetes | Physiological or imaging case-control/cross-sectional study | Human association, risk stratification, or phenotyping | JBI analytical cross-sectional | Moderate risk of bias | Selected referral samples, modest sample size, and cross-sectional design limit generalizability and causality. | Tier 2 / translational |
| 44 | Marso SP et al., 2016: Liraglutide and cardiovascular outcomes in type 2 diabetes | Randomized controlled trial | Clinical efficacy/safety | Cochrane RoB 2 | Low risk of bias | The trial enrolled patients with type 2 diabetes at high cardiovascular risk; heart-failure outcomes were secondary and do not establish HF-specific efficacy. | Tier 1 / established clinical evidence |
| 45 | Marso SP et al., 2016: Semaglutide and cardiovascular outcomes in patients with type 2 diabetes | Randomized controlled trial | Clinical efficacy/safety | Cochrane RoB 2 | Low risk of bias | The trial enrolled patients with type 2 diabetes at high cardiovascular risk; heart-failure and kidney outcomes were not the sole primary focus. | Tier 1 / established clinical evidence |
| 46 | Gerstein HC et al., 2019: Dulaglutide and cardiovascular outcomes in type 2 diabetes (REWIND): a double-blind, randomised placebo-controlled trial | Randomized controlled trial | Clinical efficacy/safety | Cochrane RoB 2 | Low risk of bias | The broad type 2 diabetes population supports cardiovascular benefit, but direct HF-specific and CKM-HF phenotype effects remain indirect. | Tier 1 / established clinical evidence |
| 47 | Zinman B et al., 2015: Empagliflozin, cardiovascular outcomes, and mortality in type 2 diabetes | Randomized controlled trial | Clinical efficacy/safety | Cochrane RoB 2 | Low risk of bias | The study involved type 2 diabetes with established cardiovascular disease; heart-failure hospitalization was a secondary outcome and mechanistic extrapolation is limited. | Tier 1 / established clinical evidence |
| 48 | Kitzman DW et al., 2016: Effect of Caloric Restriction or Aerobic Exercise Training on Peak Oxygen Consumption and Quality of Life in Obese Older Patients With Heart Failure With Preserved Ejection Fraction: A Randomized Clinical Trial | Randomized lifestyle intervention trial | Human association, risk stratification, or phenotyping | Cochrane RoB 2 | Some concerns | Small sample, unavoidable lack of participant blinding, and functional outcomes limit precision. | Tier 2 / supportive clinical evidence |
| 49 | Obokata M et al., 2017: Evidence supporting the existence of a distinct obese phenotype of heart failure with preserved ejection fraction | Physiological or imaging case-control/cross-sectional study | Human association, risk stratification, or phenotyping | JBI analytical cross-sectional | Moderate risk of bias | Selected referral samples, modest sample size, and cross-sectional design limit generalizability and causality. | Tier 2 / translational |
| 50 | Kosiborod MN et al., 2024: Semaglutide in Patients with Obesity-Related Heart Failure and Type 2 Diabetes | Randomized controlled trial | Clinical efficacy/safety | Cochrane RoB 2 | Low risk of bias | The enrolled population had obesity-related HFpEF and type 2 diabetes; findings should not be extrapolated to HFpEF without obesity or diabetes. | Tier 1 / established clinical evidence |
| 51 | Butler J et al., 2024: Semaglutide versus placebo in people with obesity-related heart failure with preserved ejection fraction: a pooled analysis of the STEP-HFpEF and STEP-HFpEF DM randomised trials | Pooled, meta-analytic, or prespecified secondary analysis | Clinical efficacy/safety | Underlying-trial RoB 2 + prespecified pooled-analysis appraisal | Moderate-to-high confidence | This pooled participant-level analysis strengthens precision but remains dependent on the design, eligibility criteria, and prespecified outcomes of the two underlying trials. | Tier 1 supportive / not independently tier-defining |
| 52 | Kuro-o M et al., 1997: Mutation of the mouse klotho gene leads to a syndrome resembling ageing | Preclinical animal/in vitro mechanistic study | Mechanistic plausibility / hypothesis generation | SYRCLE RoB (animal components) + predefined in-vitro reporting criteria | Some concerns / unclear risk | Randomization, allocation concealment, blinding, and sample-size justification are incompletely reported in many preclinical publications; human directness is limited. | Tier 3 / mechanistic or hypothesis-generating |
| 53 | Urakawa I et al., 2006: Klotho converts canonical FGF receptor into a specific receptor for FGF23 | Preclinical animal/in vitro mechanistic study | Mechanistic plausibility / hypothesis generation | SYRCLE RoB (animal components) + predefined in-vitro reporting criteria | Some concerns / unclear risk | Randomization, allocation concealment, blinding, and sample-size justification are incompletely reported in many preclinical publications; human directness is limited. | Tier 3 / mechanistic or hypothesis-generating |
| 54 | Kurosu H et al., 2006: Regulation of Fibroblast Growth Factor-23 Signaling by Klotho | Preclinical animal/in vitro mechanistic study | Mechanistic plausibility / hypothesis generation | SYRCLE RoB (animal components) + predefined in-vitro reporting criteria | Some concerns / unclear risk | Randomization, allocation concealment, blinding, and sample-size justification are incompletely reported in many preclinical publications; human directness is limited. | Tier 3 / mechanistic or hypothesis-generating |
| 55 | Shimada T et al., 2004: Targeted ablation of Fgf23 demonstrates an essential physiological role of FGF23 in phosphate and vitamin D metabolism | Preclinical animal/in vitro mechanistic study | Mechanistic plausibility / hypothesis generation | SYRCLE RoB (animal components) + predefined in-vitro reporting criteria | Some concerns / unclear risk | Randomization, allocation concealment, blinding, and sample-size justification are incompletely reported in many preclinical publications; human directness is limited. | Tier 3 / mechanistic or hypothesis-generating |
| 56 | Shimada T et al., 2004: FGF-23 is a potent regulator of vitamin D metabolism and phosphate homeostasis | Preclinical animal/in vitro mechanistic study | Mechanistic plausibility / hypothesis generation | SYRCLE RoB (animal components) + predefined in-vitro reporting criteria | Some concerns / unclear risk | Randomization, allocation concealment, blinding, and sample-size justification are incompletely reported in many preclinical publications; human directness is limited. | Tier 3 / mechanistic or hypothesis-generating |
| 57 | Shalhoub V et al., 2012: FGF23 neutralization improves chronic kidney disease-associated hyperparathyroidism yet increases mortality | Preclinical animal/in vitro mechanistic study | Mechanistic plausibility / hypothesis generation | SYRCLE RoB (animal components) + predefined in-vitro reporting criteria | Some concerns / unclear risk | Randomization, allocation concealment, blinding, and sample-size justification are incompletely reported in many preclinical publications; human directness is limited. | Tier 3 / mechanistic or hypothesis-generating |
| 58 | Grabner A et al., 2015: Activation of cardiac fibroblast growth factor receptor 4 causes left ventricular hypertrophy | Preclinical animal/in vitro mechanistic study | Mechanistic plausibility / hypothesis generation | SYRCLE RoB (animal components) + predefined in-vitro reporting criteria | Some concerns / unclear risk | Animal and cellular models support FGFR4-mediated hypertrophy, but species/model dependence and incomplete reporting of some bias safeguards limit direct clinical inference. | Tier 3 / mechanistic or hypothesis-generating |
| 59 | Gutierrez OM et al., 2008: Fibroblast growth factor 23 and mortality among patients undergoing hemodialysis | Observational cohort study | Human association, risk stratification, or phenotyping | QUIPS | Moderate risk of bias | The hemodialysis population represents advanced kidney failure; residual confounding, assay dependence, and limited generalizability to earlier CKD restrict causal inference. | Tier 2 / translational |
| 60 | Scialla JJ et al., 2014: Fibroblast growth factor-23 and cardiovascular events in CKD | Observational cohort study | Human association, risk stratification, or phenotyping | QUIPS | Moderate risk of bias | Residual confounding by kidney function, inflammation, mineral metabolism, and comorbidity remains; observational associations do not define a treatment threshold. | Tier 2 / translational |
| 61 | Ix JH et al., 2012: Fibroblast growth factor-23 and death, heart failure, and cardiovascular events in community-living individuals | Observational cohort study | Human association, risk stratification, or phenotyping | QUIPS | Moderate risk of bias | Single biomarker measurement and residual confounding limit causal inference; generalizability to advanced CKD and assay-specific thresholds is uncertain. | Tier 2 / translational |
| 62 | Hu MC et al., 2011: Klotho deficiency causes vascular calcification in chronic kidney disease | Preclinical animal/in vitro mechanistic study | Mechanistic plausibility / hypothesis generation | SYRCLE RoB (animal components) + predefined in-vitro reporting criteria | Some concerns / unclear risk | Randomization, allocation concealment, blinding, and sample-size justification are incompletely reported in many preclinical publications; human directness is limited. | Tier 3 / mechanistic or hypothesis-generating |
| 63 | Andrukhova O et al., 2014: FGF23 regulates renal sodium handling and blood pressure | Preclinical animal/in vitro mechanistic study | Mechanistic plausibility / hypothesis generation | SYRCLE RoB (animal components) + predefined in-vitro reporting criteria | Some concerns / unclear risk | Randomization, allocation concealment, blinding, and sample-size justification are incompletely reported in many preclinical publications; human directness is limited. | Tier 3 / mechanistic or hypothesis-generating |
| 64 | Vogt J et al., 2025: Regulation of alphaKlotho | Narrative/state-of-the-art review | Background/framework | SANRA (contextual appraisal only) | Contextual synthesis; not used alone to determine certainty | Secondary source; conclusions depend on the underlying primary evidence. | Contextual |
| 65 | Isakova T et al., 2011: Fibroblast Growth Factor 23 and Risks of Mortality and End-Stage Renal Disease in Patients With Chronic Kidney Disease | Observational cohort study | Human association, risk stratification, or phenotyping | QUIPS | Moderate risk of bias | Associations with mortality and kidney progression may reflect disease severity; residual confounding, reverse causation, and assay heterogeneity preclude therapeutic interpretation. | Tier 2 / translational |
| 66 | Snelson M et al., 2025: Gut-Heart Axis: The Role of Gut Microbiota and Metabolites in Heart Failure | Narrative/state-of-the-art review | Background/framework | SANRA (contextual appraisal only) | Contextual synthesis; not used alone to determine certainty | Secondary source; conclusions depend on the underlying primary evidence. | Contextual |
| 67 | Zhang Y et al., 2021: TMAO: how gut microbiota contributes to heart failure | Narrative/state-of-the-art review | Background/framework | SANRA (contextual appraisal only) | Contextual synthesis; not used alone to determine certainty | Secondary source; conclusions depend on the underlying primary evidence. | Contextual |
| 68 | Zhang Z et al., 2024: Role of the microbiota-gut-heart axis between bile acids and cardiovascular disease | Narrative/state-of-the-art review | Background/framework | SANRA (contextual appraisal only) | Contextual synthesis; not used alone to determine certainty | Secondary source; conclusions depend on the underlying primary evidence. | Contextual |
| 69 | Wang Z et al., 2011: Gut flora metabolism of phosphatidylcholine promotes cardiovascular disease | Mixed human observational and preclinical mechanistic study | Mechanistic plausibility / hypothesis generation | JBI cohort checklist (human component) + SYRCLE RoB (animal component) | Some concerns to high risk | Human association and animal mechanisms are indirect; residual confounding and preclinical reporting limitations. | Tier 3 / hypothesis-generating |
| 70 | Tang WHW et al., 2013: Intestinal microbial metabolism of phosphatidylcholine and cardiovascular risk | Prospective cohort with mechanistic components | Human association, risk stratification, or phenotyping | JBI cohort | Moderate risk of bias | Residual confounding and biomarker measurement limitations; causality not established. | Tier 2 / translational |
| 71 | Tang WHW et al., 2014: Prognostic value of elevated levels of intestinal microbe-generated metabolite trimethylamine-N-oxide in patients with heart failure | Prospective heart-failure cohort | Human association, risk stratification, or phenotyping | QUIPS | Moderate risk of bias | Residual confounding by diet, kidney clearance, congestion, and comorbidity limits causal inference; a single biomarker measurement may not capture temporal variation. | Tier 2 / translational |
| 72 | Hylemon PB et al., 2009: Bile acids as regulatory molecules | Narrative/state-of-the-art review | Background/framework | SANRA (contextual appraisal only) | Contextual synthesis; not used alone to determine certainty | Secondary source; conclusions depend on the underlying primary evidence. | Contextual |
| 73 | Koeth RA et al., 2013: Intestinal microbiota metabolism of L-carnitine, a nutrient in red meat, promotes atherosclerosis | Mixed human observational and preclinical mechanistic study | Mechanistic plausibility / hypothesis generation | JBI cohort checklist (human component) + SYRCLE RoB (animal component) | Some concerns to high risk | Human association and animal mechanisms are indirect; residual confounding and preclinical reporting limitations. | Tier 3 / hypothesis-generating |
| 74 | Organ CL et al., 2016: Choline diet and its gut microbe-derived metabolite, trimethylamine N-oxide, exacerbate pressure overload-induced heart failure | Preclinical animal/in vitro mechanistic study | Mechanistic plausibility / hypothesis generation | SYRCLE RoB (animal components) + predefined in-vitro reporting criteria | Some concerns / unclear risk | Randomization, allocation concealment, blinding, and sample-size justification are incompletely reported in many preclinical publications; human directness is limited. | Tier 3 / mechanistic or hypothesis-generating |
| 75 | David LA et al., 2014: Diet rapidly and reproducibly alters the human gut microbiome | Small controlled dietary intervention/crossover study | Mechanistic plausibility / hypothesis generation | JBI quasi-experimental | High risk of bias / serious limitations | Very small sample, short exposure, and limited clinical outcomes. | Tier 3 / hypothesis-generating |
| 76 | Wahlstrom A et al., 2016: Intestinal crosstalk between bile acids and microbiota and its impact on host metabolism | Narrative/state-of-the-art review | Background/framework | SANRA (contextual appraisal only) | Contextual synthesis; not used alone to determine certainty | Secondary source; conclusions depend on the underlying primary evidence. | Contextual |
| 77 | Grabner A et al., 2017: FGF23/FGFR4-mediated left ventricular hypertrophy is reversible | Preclinical animal/in vitro mechanistic study | Mechanistic plausibility / hypothesis generation | SYRCLE RoB (animal components) + predefined in-vitro reporting criteria | Some concerns / unclear risk | Reversibility was demonstrated in preclinical models, but human safety, target specificity, phosphate consequences, and clinical outcome benefits remain untested. | Tier 3 / mechanistic or hypothesis-generating |
| 78 | McMurray JJV et al., 2019: Dapagliflozin in patients with heart failure and reduced ejection fraction | Randomized controlled trial | Clinical efficacy/safety | Cochrane RoB 2 | Low risk of bias | Directness is limited to the enrolled phenotype and prespecified outcomes; subgroup/mechanistic extrapolation requires caution. | Tier 1 / established clinical evidence |
| 79 | Packer M et al., 2020: Cardiovascular and renal outcomes with empagliflozin in heart failure | Randomized controlled trial | Clinical efficacy/safety | Cochrane RoB 2 | Low risk of bias | Directness is limited to the enrolled phenotype and prespecified outcomes; subgroup/mechanistic extrapolation requires caution. | Tier 1 / established clinical evidence |
| 80 | Perkovic V et al., 2019: Canagliflozin and renal outcomes in type 2 diabetes and nephropathy | Randomized controlled trial | Clinical efficacy/safety | Cochrane RoB 2 | Low risk of bias | Directness is limited to the enrolled phenotype and prespecified outcomes; subgroup/mechanistic extrapolation requires caution. | Tier 1 / established clinical evidence |
| 81 | Wiviott SD et al., 2019: Dapagliflozin and cardiovascular outcomes in type 2 diabetes | Randomized controlled trial | Clinical efficacy/safety | Cochrane RoB 2 | Low risk of bias | Directness is limited to the enrolled phenotype and prespecified outcomes; subgroup/mechanistic extrapolation requires caution. | Tier 1 / established clinical evidence |
| 82 | Neal B et al., 2017: Canagliflozin and cardiovascular and renal events in type 2 diabetes | Randomized controlled trial | Clinical efficacy/safety | Cochrane RoB 2 | Low risk of bias | Directness is limited to the enrolled phenotype and prespecified outcomes; subgroup/mechanistic extrapolation requires caution. | Tier 1 / established clinical evidence |
| 83 | Zannad F et al., 2020: SGLT2 inhibitors in patients with heart failure with reduced ejection fraction: a meta-analysis of the EMPEROR-Reduced and DAPA-HF trials | Pooled, meta-analytic, or prespecified secondary analysis | Clinical efficacy/safety | AMSTAR 2 + underlying-trial RoB 2 | Moderate-to-high confidence | The meta-analysis included a limited number of major trials and inherits their eligibility criteria; subgroup and mechanistic conclusions remain less certain than the pooled HF outcome. | Tier 1 supportive / not independently tier-defining |
| 84 | Cannon CP et al., 2020: Cardiovascular outcomes with ertugliflozin in type 2 diabetes | Randomized controlled trial | Clinical efficacy/safety | Cochrane RoB 2 | Low risk of bias | Directness is limited to the enrolled phenotype and prespecified outcomes; subgroup/mechanistic extrapolation requires caution. | Tier 1 / established clinical evidence |
| 85 | Bhatt DL et al., 2021: Sotagliflozin in patients with diabetes and recent worsening heart failure | Randomized controlled trial | Clinical efficacy/safety | Cochrane RoB 2 | Some concerns | Early trial termination and protocol/endpoint changes reduce certainty, although randomization was preserved. | Tier 1 / established clinical evidence |
| 86 | Bhatt DL et al., 2021: Sotagliflozin in patients with diabetes and chronic kidney disease | Randomized controlled trial | Clinical efficacy/safety | Cochrane RoB 2 | Some concerns | Early trial termination and protocol/endpoint changes reduce certainty, although randomization was preserved. | Tier 1 / established clinical evidence |
| 87 | Lincoff AM et al., 2023: Semaglutide and cardiovascular outcomes in obesity without diabetes | Randomized controlled trial | Clinical efficacy/safety | Cochrane RoB 2 | Low risk of bias | Directness is limited to the enrolled phenotype and prespecified outcomes; subgroup/mechanistic extrapolation requires caution. | Tier 1 / established clinical evidence |
| 88 | Jastreboff AM et al., 2022: Tirzepatide once weekly for the treatment of obesity | Randomized controlled trial | Clinical efficacy/safety | Cochrane RoB 2 | Low risk of bias | Directness is limited to the enrolled phenotype and prespecified outcomes; subgroup/mechanistic extrapolation requires caution. | Tier 1 / established clinical evidence |
| 89 | Wilding JPH et al., 2021: Once-weekly semaglutide in adults with overweight or obesity | Randomized controlled trial | Clinical efficacy/safety | Cochrane RoB 2 | Low risk of bias | Directness is limited to the enrolled phenotype and prespecified outcomes; subgroup/mechanistic extrapolation requires caution. | Tier 1 / established clinical evidence |
| 90 | Rubino D et al., 2021: Effect of Continued Weekly Subcutaneous Semaglutide vs Placebo on Weight Loss Maintenance in Adults With Overweight or Obesity: The STEP 4 Randomized Clinical Trial | Randomized withdrawal trial | Clinical efficacy/safety | Cochrane RoB 2 | Some concerns | Enriched responder population and withdrawal design limit generalizability. | Tier 1 / supportive clinical evidence |
| 91 | Holman RR et al., 2017: Effects of once- weekly exenatide on cardiovascular outcomes in type 2 diabetes | Pragmatic open-label randomized trial | Clinical efficacy/safety | Cochrane RoB 2 | Some concerns | Open-label treatment and pragmatic follow-up may influence adherence and outcome ascertainment. | Tier 1 / established clinical evidence |
| 92 | Husain M et al., 2019: Oral semaglutide and cardiovascular outcomes in patients with type 2 diabetes | Randomized controlled trial | Clinical efficacy/safety | Cochrane RoB 2 | Low risk of bias | Directness is limited to the enrolled phenotype and prespecified outcomes; subgroup/mechanistic extrapolation requires caution. | Tier 1 / established clinical evidence |
| 93 | Gerstein HC et al., 2021: Cardiovascular and renal outcomes with efpeglenatide in type 2 diabetes | Randomized controlled trial | Clinical efficacy/safety | Cochrane RoB 2 | Low risk of bias | Directness is limited to the enrolled phenotype and prespecified outcomes; subgroup/mechanistic extrapolation requires caution. | Tier 1 / established clinical evidence |
| 94 | Mann JFE et al., 2017: Liraglutide and renal outcomes in type 2 diabetes | Pooled, meta-analytic, or prespecified secondary analysis | Clinical efficacy/safety | Underlying-trial RoB 2 + outcome-specific secondary-analysis appraisal | Moderate-to-high confidence | Renal outcomes were secondary analyses of the parent cardiovascular trial and may be more vulnerable to multiplicity and indirectness than the primary endpoint. | Tier 1 supportive / not independently tier-defining |
| 95 | Tuttle KR et al., 2018: Dulaglutide versus insulin glargine in patients with type 2 diabetes and moderate-to-severe chronic kidney disease (AWARD-7): a multicentre, open-label, randomised trial | Open-label randomized controlled trial | Clinical efficacy/safety | Cochrane RoB 2 | Some concerns | Open-label design and renal secondary outcomes limit certainty. | Tier 1 / supportive clinical evidence |
| 96 | Pitt B et al., 2014: Spironolactone for heart failure with preserved ejection fraction | Randomized controlled trial | Clinical efficacy/safety | Cochrane RoB 2 | Some concerns | Regional heterogeneity, adherence concerns, and a neutral primary endpoint complicate interpretation. | Tier 1 / supportive clinical evidence |
| 97 | Solomon SD et al., 2019: Angiotensin-neprilysin inhibition in heart failure with preserved ejection fraction | Randomized controlled trial | Clinical efficacy/safety | Cochrane RoB 2 | Low risk of bias | Directness is limited to the enrolled phenotype and prespecified outcomes; subgroup/mechanistic extrapolation requires caution. | Tier 1 / established clinical evidence |
| 98 | Agarwal R et al., 2022: Cardiovascular and kidney outcomes with finerenone in patients with type 2 diabetes and chronic kidney disease: the FIDELITY pooled analysis | Pooled, meta-analytic, or prespecified secondary analysis | Clinical efficacy/safety | Underlying-trial RoB 2 + prespecified pooled-analysis appraisal | Moderate-to-high confidence | The pooled analysis increases precision but remains dependent on two closely related trials in albuminuric type 2 diabetes with CKD and does not independently establish broader generalizability. | Tier 1 supportive / not independently tier-defining |
| 99 | Wang TJ et al., 2004: Impact of Obesity on Plasma Natriuretic Peptide Levels | Observational cohort study | Human association, risk stratification, or phenotyping | QUIPS | Moderate risk of bias | Natriuretic peptide concentrations are influenced by age, sex, kidney function, rhythm, and HF phenotype; residual confounding limits causal interpretation. | Tier 2 / translational |
| 100 | Lang RM et al., 2015: Recommendations for Cardiac Chamber Quantification by Echocardiography in Adults: An Update from the American Society of Echocardiography and the European Association of Cardiovascular Imaging | Expert consensus/recommendation statement | Guideline/measurement standard | AGREE II-GRS | Moderate-to-high methodological confidence | Consensus-based elements and indirect evidence remain. | Tier 1 for diagnostic/measurement standards |
| 101 | Chertow GM et al., 2012: Effect of cinacalcet on cardiovascular disease in patients undergoing dialysis (EVOLVE) | Randomized controlled outcome trial | Clinical efficacy/safety and cardiovascular-outcome evidence for CKD-MBD intervention | Cochrane RoB 2 | Some concerns | High treatment discontinuation and crossover reduced treatment separation; the prespecified unadjusted intention-to-treat primary outcome was neutral, and adjusted analyses were secondary. The intervention was cinacalcet rather than a phosphate binder and was not HF-specific. | Tier 1 / randomized clinical outcome evidence |
| 102 | Block GA et al., 2012: Effects of phosphate binders in moderate CKD | Randomized placebo-controlled trial | Supportive clinical evidence on phosphate lowering, FGF23, and vascular calcification | Cochrane RoB 2 | Some concerns | Modest sample size, short follow-up, normal or near-normal baseline phosphate, heterogeneous binder classes, and reliance on biochemical and vascular-calcification surrogate outcomes limit clinical-outcome inference. | Tier 2 / supportive clinical evidence |
| 103 | Toussaint ND et al., 2020: A randomized trial on the effect of phosphate reduction on vascular end points in CKD (IMPROVE-CKD) | Randomized placebo-controlled trial | Supportive clinical evidence on phosphate lowering and vascular surrogate outcomes | Cochrane RoB 2 | Some concerns | The trial evaluated arterial stiffness and aortic calcification rather than HF or cardiovascular events; stage 3b–4 CKD eligibility, modest sample size, treatment adherence, and surrogate endpoints limit generalizability and precision. | Tier 2 / supportive clinical evidence |
| 104 | Ogata H et al., 2021: Effect of treating hyperphosphatemia with lanthanum carbonate vs calcium carbonate on cardiovascular events in patients undergoing hemodialysis (LANDMARK) | Randomized active-comparator cardiovascular outcome trial | Clinical efficacy/safety and cardiovascular-outcome evidence for phosphate-binder therapy | Cochrane RoB 2 | Some concerns | Open-label treatment, active-comparator design, dialysis-specific population, and absence of superiority limit extrapolation to earlier CKD, placebo effects, HF-specific outcomes, or FGF23-guided treatment. | Tier 1 / randomized clinical outcome evidence |
| 105 | Panwar B et al., 2018: Association of Fibroblast Growth Factor 23 With Risk of Incident Coronary Heart Disease in Community-Living Adults | Prospective case-cohort study within the REGARDS cohort | Human association and sex-specific biomarker interpretation | QUIPS | Moderate risk of bias | Observational case-cohort design with a single baseline C-terminal FGF23 measurement limits causal inference. The sex interaction was borderline, sex-stratified estimates were sensitive to sex-specific biomarker distributions and adjustment for hormone therapy, and some subgroup estimates were imprecise. Residual confounding remains, serum phosphate and calcium and contemporary cardiac biomarkers were unavailable, and generalizability beyond Black and White US adults is limited. | Tier 2 / translational |
| 106 | Swartling O et al., 2021: CKD Progression and Mortality Among Men and Women: A Nationwide Study in Sweden | Nationwide registry-based observational cohort study | Human association and sex-specific CKD progression and mortality | QUIPS | Moderate risk of bias | Large national registry and competing-risk analyses strengthen precision, but the observational design cannot establish biological causality. The cohort was restricted to nephrology-referred incident CKD G3b-G5; outpatient visits and laboratory data were incomplete, albuminuria data were limited, and regional reporting varied. Residual confounding, differential referral or treatment, binary recording of sex, and limited generalizability outside the Swedish health system remain. | Tier 2 / translational |
| 107 | Wang X et al., 2023: Sex Differences in Characteristics, Outcomes, and Treatment Response With Dapagliflozin Across the Range of Ejection Fraction in Patients With Heart Failure: Insights From DAPA-HF and DELIVER | Prespecified patient-level pooled secondary analysis of two randomized controlled trials | Sex-specific treatment response, outcomes, and safety | Underlying-trial RoB 2 + prespecified pooled-analysis appraisal | Moderate-to-high confidence | The analysis was prespecified and based on two low-risk randomized trials, but it remains a secondary subgroup analysis and was not independently powered to exclude small treatment-by-sex interactions. Women comprised 35% of participants, sex was analyzed in binary categories, gender identity was not reported, and applicability is limited by the parent trials' eligibility criteria and outcome definitions. The analysis does not establish sex-specific FGF23-Klotho mediation. | Tier 1 supportive / not independently tier-defining |

**Notes:** DS and XW independently appraised each evidence-generating publication using design-specific instruments and resolved disagreements through discussion until consensus. Judgments were not converted into a common numerical score because the appraisal tools use different domains and categories.

Evidence tiers reflected clinical directness and actionability after consideration of study design, risk of bias, consistency, and applicability: Tier 1 = established or practice-informing clinical evidence; Tier 2 = human translational, observational, prognostic, biomarker, physiological, or supportive clinical evidence; Tier 3 = mechanistic or hypothesis-generating evidence; Contextual = background source not independently tier-defining. Methodological quality and evidence tier were not treated as synonymous.

**Abbreviations:** AGREE II, Appraisal of Guidelines for Research and Evaluation II; AMSTAR 2, A MeaSurement Tool to Assess systematic Reviews 2; JBI, Joanna Briggs Institute; PROBAST, Prediction model Risk Of Bias ASsessment Tool; QUIPS, Quality In Prognosis Studies; RoB 2, Risk of Bias 2; SANRA, Scale for the Assessment of Narrative Review Articles; SYRCLE, Systematic Review Centre for Laboratory animal Experimentation.

**Methodological references:** Cochrane Methods. RoB 2: a revised Cochrane risk-of-bias tool for randomized trials; see Cochrane Handbook, Chapter 8. JBI. Critical Appraisal Tools for cohort, case-control, analytical cross-sectional, and quasi-experimental studies. Hayden JA, van der Windt DA, Cartwright JL, Côté P, Bombardier C. Assessing bias in studies of prognostic factors. Ann Intern Med. 2013;158:280–286. doi:10.7326/0003-4819-158-4-201302190-00009. Wolff RF, Moons KGM, Riley RD, et al. PROBAST: A Tool to Assess the Risk of Bias and Applicability of Prediction Model Studies. Ann Intern Med. 2019;170:51–58. doi:10.7326/M18-1376. Brouwers MC, Kho ME, Browman GP, et al. AGREE II: advancing guideline development, reporting and evaluation in health care. CMAJ. 2010;182:E839–E842. doi:10.1503/cmaj.090449. Hooijmans CR, Rovers MM, de Vries RBM, Leenaars M, Ritskes-Hoitinga M, Langendam MW. SYRCLE’s risk of bias tool for animal studies. BMC Med Res Methodol. 2014;14:43. doi:10.1186/1471-2288-14-43. Shea BJ, Reeves BC, Wells G, et al. AMSTAR 2: a critical appraisal tool for systematic reviews that include randomized or non-randomized studies of healthcare interventions, or both. BMJ. 2017;358:j4008. doi:10.1136/bmj.j4008. Baethge C, Goldbeck-Wood S, Mertens S. SANRA—a scale for the quality assessment of narrative review articles. Res Integr Peer Rev. 2019;4:5. doi:10.1186/s41073-019-0064-8. Balshem H, Helfand M, Schünemann HJ, et al. GRADE guidelines: 3. Rating the quality of evidence. J Clin Epidemiol. 2011;64:401–406. doi:10.1016/j.jclinepi.2010.07.015.
