# Supplementary material for: Endocrine crosstalk in cardiovascular-kidney-metabolic syndrome-related heart failure: the fibroblast growth factor 23-Klotho axis and cardiometabolic therapy
Source: Front Endocrinol (Lausanne). 2026 Sep 8;17:1933732. doi: 10.3389/fendo.2026.1933732 (PMC13594805; doi:10.3389/fendo.2026.1933732)
Supplement: Supplementary file 3 [file Table3.docx]

**Supplementary Table S3.** GRADE-informed certainty assessment for major clinical and translational propositions.

| **Clinical or translational proposition** | **Supporting references** | **Evidence base** | **Risk of bias** | **Inconsistency** | **Indirectness** | **Imprecision** | **Publication bias** | **Overall certainty** | **Evidence tier** | **Permitted manuscript wording** |
| --- | --- | --- | --- | --- | --- | --- | --- | --- | --- | --- |
| SGLT2 inhibitors reduce heart-failure events across HFrEF, HFmrEF, and HFpEF populations. | 20, 25, 78, 79, 83, 85 | Multiple large randomized outcome trials and a trial-level meta-analysis | Not serious overall; some concerns for early-terminated SOLOIST-WHF | Not serious | Not serious for HF outcomes; phenotype-specific extrapolation should remain explicit | Not serious | Not formally assessed; not strongly suspected | High | Tier 1 | reduces HF events / supports clinical use in eligible patients |
| SGLT2 inhibitors improve kidney outcomes in CKD, including populations with and without diabetes. | 22, 38, 80, 82, 86 | Multiple large randomized kidney and cardiovascular outcome trials | Not serious overall; some concerns for early-terminated SCORED | Not serious | Not serious | Not serious | Not formally assessed; not strongly suspected | High | Tier 1 | improves kidney outcomes / reduces progression in eligible CKD populations |
| Finerenone reduces kidney and cardiovascular events in patients with type 2 diabetes and CKD. | 21, 24, 98 | Two large randomized trials plus pooled analysis | Not serious | Not serious | Not serious for albuminuric T2D-CKD | Not serious | Not formally assessed; not strongly suspected | High | Tier 1 | reduces kidney and cardiovascular events in the studied population |
| Finerenone reduces worsening heart failure or cardiovascular death in HFmrEF/HFpEF. | 37 | One large randomized outcome trial | Not serious | Cannot be fully assessed from a single pivotal trial | Not serious for the enrolled HFmrEF/HFpEF population | Not serious | Not formally assessed; cannot be excluded | Moderate | Tier 1 | supports benefit in the studied HFmrEF/HFpEF population |
| Semaglutide improves symptoms, physical limitations, and body weight in obesity-related HFpEF. | 23, 50, 51 | Two randomized trials and pooled participant-level analysis | Not serious | Not serious | Restricted to obesity-related HFpEF, with and without T2D | Not serious | Not formally assessed; not strongly suspected | High | Tier 1 | improves symptoms, function, and weight in obesity-related HFpEF |
| GLP-1 receptor agonist-based therapy reduces cardiovascular and/or kidney events in selected T2D, CKD, or obesity populations. | 36, 44–46, 87, 91–95 | Multiple randomized cardiovascular, kidney, and obesity outcome trials | Not serious overall; some concerns in open-label or secondary renal analyses | Not serious for broad cardiovascular benefit; renal endpoints vary by trial | Some indirectness when extrapolated specifically to HF outcomes | Not serious | Not formally assessed; not strongly suspected | High for cardiovascular outcomes; Moderate for direct HF-specific inference | Tier 1 | improves cardiovascular/kidney outcomes in selected populations; HF-specific effects should be qualified |
| Caloric restriction and aerobic exercise improve exercise capacity and quality of life in obese older patients with HFpEF. | 48 | Single small randomized lifestyle trial | Some concerns | Cannot be assessed | Not serious for the selected obese older HFpEF population | Serious because of sample size and functional endpoints | Not formally assessable | Low | Tier 2 | may improve exercise capacity and quality of life |
| Lower eGFR and higher albuminuria are associated with greater cardiovascular and mortality risk. | 29, 30 | Large observational cohort and collaborative meta-analysis | Serious residual confounding inherent to observational evidence | Not serious | Not serious | Not serious | Not formally assessed; unlikely to explain the graded associations | Moderate | Tier 2 / established epidemiologic association | are strongly associated with cardiovascular and mortality risk |
| Higher circulating FGF23 is associated with LVH, incident HF, HF subtype, mortality, and kidney progression. | 18, 33–35, 59–61, 65 | Cross-sectional and prospective observational biomarker studies | Serious because of residual confounding, assay heterogeneity, and reverse causation | Some inconsistency across populations and endpoints | Not serious for association; serious for therapeutic actionability | Generally not serious, but subtype analyses are less precise | Not formally assessed; possible | Low | Tier 2 | is associated with / may help phenotype; does not establish causality or a treatment threshold |
| Preclinical studies suggest that FGF23 may promote cardiac hypertrophy through FGFR4-related signaling. | 17, 58, 77 | Animal, cellular, and preclinical intervention studies | Some concerns/unclear because of incomplete reporting of randomization and blinding | Not serious within the limited preclinical literature | Very serious for inference to human cardiac remodeling and clinical outcomes | Serious | Not formally assessed; possible | Very low | Tier 3 | supports biological plausibility in preclinical models / may promote hypertrophic signaling; direct causality in humans is not established |
| Preclinical and translational evidence suggests that Klotho deficiency may contribute to mineral dysregulation, vascular calcification, and cardiovascular vulnerability; therapeutic Klotho restoration remains investigational. | 52–64 | Predominantly animal, cellular, and physiological studies plus narrative synthesis | Some concerns/unclear in preclinical studies | Some inconsistency in assays and experimental contexts | Very serious for clinical biomarker thresholds and treatment | Serious | Not formally assessed; possible | Very low | Tier 3 | may contribute / remains investigational; routine clinical use is not established |
| Phosphate-lowering and related CKD-MBD interventions have not consistently reduced cardiovascular events in CKD or dialysis populations. | 39, 101–104 | CKD-MBD guideline plus randomized trials of cinacalcet and phosphate-binder strategies across nondialysis CKD and hemodialysis populations | Some concerns overall because of treatment discontinuation or crossover in EVOLVE and open-label, adherence, or surrogate-endpoint limitations in some binder trials | Some concern because interventions, CKD stages, comparators, and cardiovascular or vascular endpoints differ substantially | Serious for HF-specific inference; several studies evaluated mineral-metabolism interventions or vascular surrogate endpoints rather than HF events | Some concerns because several studies were modest in size and event or surrogate estimates were not uniformly precise | Not formally assessed; possible | Low | Tier 1 for randomized outcome evidence; Tier 2 for supportive surrogate evidence | has not consistently reduced cardiovascular events / cardiovascular benefit remains unproven; use phosphate-lowering therapy for established CKD-MBD indications rather than HF prevention or FGF23-guided treatment |
| TMAO and related gut-derived metabolites are associated with cardiovascular and HF risk, but causal and therapeutic relevance remain uncertain. | 69–75 | Human observational biomarker studies, small dietary studies, and animal experiments | Serious residual confounding and preclinical reporting limitations | Some inconsistency and strong dependence on diet and kidney clearance | Serious for treatment decisions | Serious for mechanistic and interventional claims | Not formally assessed; possible | Low for association; Very low for causality/intervention | Tier 2 for association; Tier 3 for mechanism | is associated with / may contribute; not ready for routine clinical stratification |
| HFpEF includes a systemic cardiometabolic and microvascular phenotype rather than a single uniform myocardial disorder. | 10–13, 43, 49 | Population cohorts, invasive physiology, imaging, metabolic, and autopsy studies | Moderate risk across observational studies | Not serious for heterogeneity; specific mechanisms vary | Not serious for phenotype description; serious for individual treatment selection | Some concerns in small mechanistic cohorts | Not formally assessed; possible | Low to moderate | Tier 2 | supports a heterogeneous systemic phenotype / may inform phenotyping |
| Sex-related differences may influence HFpEF presentation, CKD progression, and circulating FGF23 associations, but current evidence does not support sex-specific FGF23 or soluble Klotho thresholds. | 6, 10, 105, 106 | HFpEF epidemiologic synthesis plus prospective community case-cohort and nationwide registry-based observational cohorts | Serious because of residual confounding, single biomarker measurement, selection or referral effects, and binary sex classification | Some concerns because the direction and magnitude of sex-related differences vary by population, endpoint, and adjustment strategy | Serious for CKM-HF-specific biomarker thresholds and treatment decisions; gender identity was not evaluated | Some concerns for interaction and sex-stratified estimates | Not formally assessed; possible | Low | Tier 2 | may differ by sex / should be examined using sex-disaggregated analyses; does not support sex-specific biomarker thresholds or treatment algorithms |
| Clinical benefits of dapagliflozin appear broadly consistent in women and men across the ejection-fraction spectrum. | 25, 78, 107 | Two large randomized outcome trials plus a prespecified participant-level pooled sex analysis | Not serious for the parent trials; some concerns because the sex analysis was secondary and not independently powered to exclude small interactions | Not serious; no material treatment-by-sex interaction was identified across the pooled trials | Not serious for dapagliflozin in the enrolled HF populations; serious for extrapolation to other therapies, FGF23-Klotho mediation, or gender-diverse populations | Some concerns because women represented 35% of participants and small differential effects cannot be excluded | Not formally assessed; not strongly suspected | Moderate | Tier 1 supportive / not independently tier-defining | benefit appears broadly consistent in women and men; does not establish equivalence or justify sex-specific treatment algorithms |

**Notes:** DS and XW independently assessed each proposition-specific body of evidence and resolved disagreements through discussion until consensus. Certainty was assigned to a proposition- or outcome-specific body of evidence rather than to an individual publication. The term “GRADE-informed” was used because the review integrates intervention, prognostic, biomarker, physiological, and preclinical evidence rather than a single prespecified meta-analytic question. For sex- and gender-related propositions, the available evidence predominantly used binary sex categories; gender identity was generally not recorded and could not be separately assessed.

Evidence tiers describe clinical directness and actionability and are distinct from certainty. A high-quality preclinical experiment remains Tier 3 because it is indirect to patient outcomes. Publication bias was not formally quantified because the propositions combined heterogeneous designs and outcomes; judgments therefore considered trial registration, the size and completeness of the evidence base, and the plausibility of selective publication.

**Certainty definitions:** High = high confidence that the proposition is correct for the specified population and outcome; Moderate = the proposition is likely correct, but new evidence could materially change the estimate or interpretation; Low = confidence is limited because evidence is observational, heterogeneous, confounded, indirect, or imprecise; Very low = the proposition is principally mechanistic, preclinical, assay-dependent, highly indirect, or methodologically limited.

**Methodological references**

Cochrane Methods. RoB 2: a revised Cochrane risk-of-bias tool for randomized trials; see Cochrane Handbook, Chapter 8.

JBI. Critical Appraisal Tools for cohort, case-control, analytical cross-sectional, and quasi-experimental studies.

Hayden JA, van der Windt DA, Cartwright JL, Côté P, Bombardier C. Assessing bias in studies of prognostic factors. Ann Intern Med. 2013;158:280–286. doi:10.7326/0003-4819-158-4-201302190-00009.

Wolff RF, Moons KGM, Riley RD, et al. PROBAST: A Tool to Assess the Risk of Bias and Applicability of Prediction Model Studies. Ann Intern Med. 2019;170:51–58. doi:10.7326/M18-1376.

Brouwers MC, Kho ME, Browman GP, et al. AGREE II: advancing guideline development, reporting and evaluation in health care. CMAJ. 2010;182:E839–E842. doi:10.1503/cmaj.090449.

Hooijmans CR, Rovers MM, de Vries RBM, Leenaars M, Ritskes-Hoitinga M, Langendam MW. SYRCLE’s risk of bias tool for animal studies. BMC Med Res Methodol. 2014;14:43. doi:10.1186/1471-2288-14-43.

Shea BJ, Reeves BC, Wells G, et al. AMSTAR 2: a critical appraisal tool for systematic reviews that include randomized or non-randomized studies of healthcare interventions, or both. BMJ. 2017;358:j4008. doi:10.1136/bmj.j4008.

Baethge C, Goldbeck-Wood S, Mertens S. SANRA—a scale for the quality assessment of narrative review articles. Res Integr Peer Rev. 2019;4:5. doi:10.1186/s41073-019-0064-8.

Balshem H, Helfand M, Schünemann HJ, et al. GRADE guidelines: 3. Rating the quality of evidence. J Clin Epidemiol. 2011;64:401–406. doi:10.1016/j.jclinepi.2010.07.015.
